# Thyroid hormone synthesis: a potential target of a Chinese herbal formula Haizao Yuhu Decoction acting on iodine-deficient goiter

**DOI:** 10.18632/oncotarget.10329

**Published:** 2016-06-30

**Authors:** Yanqiong Zhang, Yuting Li, Xia Mao, Chen Yan, Xiaodong Guo, Qiuyan Guo, Zhenli Liu, Zhiqian Song, Na Lin

**Affiliations:** ^1^ Institute of Chinese Materia Medica, China Academy of Chinese Medical Sciences, Beijing 100700, China; ^2^ 302 Hospital of PLA, Beijing 100039, China; ^3^ Institute of Basic Theory, China Academy of Chinese Medical Sciences, Beijing 100700, China

**Keywords:** Chinese herbal formula, goiter, genome-wide microarray, network pharmacology, experimental validation

## Abstract

Haizao Yuhu Decoction (HYD), a famous multi-component herbal formula, has been widely used to treat various thyroid-related diseases, including iodine-deficient goiter. Herb pair *Thallus Sargassi Pallidi* (HZ) and *Radix Glycyrrhizae* (GC), one of the so-called “eighteen antagonistic medicaments”, contains in HYD. To explore pharmacological mechanisms of HYD acting on iodine-deficient goiter and to provide evidence for potential roles of herb pair HZ and GC in HYD, our genome-wide microarray detection and network analysis identified a list of goiter-related genes, mainly involved into the alterations in hypothalamus-pituitary-thyroid/gonad/growth axes. Then, the disease genes-drug genes interaction network illustrated the links between HYD regulating genes and goiter-related genes, and identified the candidate targets of HYD acting on goiter. Functionally, these candidate targets were closely correlated with thyroid hormone synthesis. Moreover, the potential regulating genes of herb pair HZ and GC were revealed to be crucial components in the pathway of thyroid hormone synthesis. The prediction results were all verified by following experiments based on goiter rats. Collectively, this integrative study combining microarray gene expression profiling, network analysis and experimental validations offers the convincing evidence that HYD may alleviate iodine-deficient goiter via regulating thyroid hormone synthesis, and explains the necessity of herb pair HZ and GC in HYD. Our work provides a novel and powerful means to clarify the mechanisms of action for multi-component drugs such as herbal formulae in a holistic way, which may improve drug development and applications.

## INTRODUCTION

Goiter represents thyroid pathological enlargement, which is characterized by the cellular proliferation of thyroid follicular cells with simultaneous pathological changes including the neo-angiogenesis, the accumulation and involution of colloid, and the hyperplasia of connective tissues [1]. In clinics, signs of goiter are only visible when the compression of important structures in the neck occurs, and are often accompanied by the blockage in trachea, subclavia arterial/venous flow, esophagus or thoracic cavity, leading to the disfigurement of these tissues, and even dyspnea, stridor, cough, and choking sensation [2]. Iodine deficiency has been recognized as the most important risk factor for goiter worldwide [3]. Growing evidence show a close correlation between iodine intake and the incidence of goiter [4]. With advances in L-T_4_ suppressive therapy, surgery and ^131^I therapy, the clinical cure rate for goiter has been greatly improved. These therapeutic strategies have advantages of low cost, new nodule formation prevention, marked goiter reduction and improvement in inspiratory capacity [5]. However, several drawbacks and limitations exist in these administrations, including adverse events in bone and heart, hypothyroidism and recurrence dependent of resection, as well as increased risks of vocal cord paralysis, hypoparathyroidism and further transformation into Graves' disease and even cancer [6–10]. Therefore, it is of great clinical significance to understand the molecular mechanisms involving goiter development and progression, which will be necessary to develop more efficient therapeutic strategies for patients with goiter.

Traditional Chinese Medicine (TCM), an essential component of the current medical system, has been extensively applied in clinical practice because of its valuable therapeutic efficacy with few adverse effects. There are various Chinese herbal formulae clinically prescribed to cure goiter by invigorating blood circulation, especially microcirculation, eliminating phlegm, and softening and resolving masses [11]. Haizao Yuhu Decoction (HYD), as a classic TCM formula originally described in “Waike Zhengzong (Summary of Surgical Medicine) in Ming dynasty”, has been used to treat goiter for approximately 500 years. It is prepared from a basic formula consisting *Thallus Sargassi Pallidi* (Hai Zao, HZ), *Thallus Laminariae Japonicae* (Kun Bu, KB), *Rhizoma Pinelliae Preparata* (Fa Ban Xia, FBX), *Bulbus Fritillariae Thunbergii* (Zhe Bei Mu, ZBM), *Pericarpium Citri Reticulatae* (Qing Pi, QP), *Pericarpium Citrus reticulata* (Cheng Pi, CP), *Radix Angelicae Sinensis* (Dang Gui, DG), *Rhizoma Chanxiong* (Chuan Xiong, CX), *Radix Angelicae Pubescentis* (Du Huo, DH), *Fructus Forsythiae Suspensae* (Lian Qiao, LQ) and *Radix Glycyrrhiza* (Gan Cao, GC), and is widely produced in China in accordance with the China Pharmacopoeia standard of quality control. HYD has prominent therapeutic effects in resolving “*qi*” stagnation and phlegm coagulation [12]. Moreover, because it is rich in iodine, approximately 90% of patients with iodine-deficient goiter may achieve clinical remission after the administration of HYD without any significant side effects [13, 14]. Especially, HYD treatment has been indicated to increase the production of reactive oxygen species, enhance the anti-oxidative activity, and decrease the lipid peroxidation in thyroid tissues, which may reduce the damage of excessive iodine to this organ [15, 16]. Notably, the herb pair HZ and GC is indicated as one of the so-called “eighteen antagonistic medicaments” in Chinese medicinal literature, implying that the two herbs are mutually incompatible and thus theoretically should not be applied simultaneously. However, the HZ and GC combination is prescribed in HYD. Therefore, it is necessary to investigate the rationality of the herbaceous compatibilities between HZ and GC in HYD.

Chinese herbal formulae are complex mixtures of herbs consisting of multiple bioactive ingredients, which bind to the corresponding targets simultaneously, transiently, or weakly, and in combination to treat complex diseases in a systematic manner. Due to their complex nature and the lack of scientific methods, the molecular mechanisms of Chinese herbal formulae remain elusive. Novel and efficient approaches are urgently demanded for identifying mutual interactions and further interpreting the scientific basis of TCM formula. With the rapid advancement in bioinformatics, systems biology and polypharmacology, “network pharmacology” [17–18], shifting the “one target, one drug” paradigm to the “network target, multi-component” strategy [19], has attracted more attentions of TCM researchers, because it can not only reveal the underlying complex interactions between a herbal formula and cellular proteins, but detect the influence of their interactions on the function and behavior of the human system, of which the key idea is in line with the holistic theory of TCM. Our recent studies have applied network pharmacology-based approaches to successfully clarify the synergistic effects among herbs and investigate molecular mechanisms of various herbal formulae, such as Wu-Tou decoction [20], Gansui Banxia decoction [21], Buyang Huanwu decoction [22] and Guizhi Shaoyao Zhimu decoction [23].

**Figure 1:**
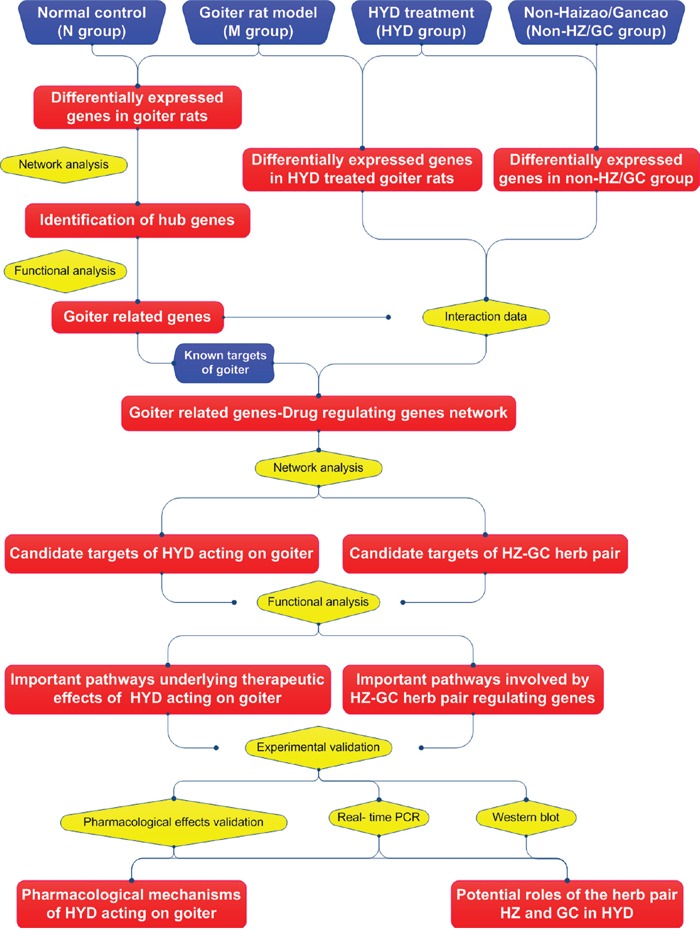
A schematic diagram of the integrative strategy combining microarray gene expression profiling, network analysis and experimental validations for investigating the pharmacological mechanisms of herbal formula Haizao Yuhu Decoction acting on iodine-deficient goiter

To explore the pharmacological mechanisms of HYD acting on iodine-deficient goiter and to provide evidence for the potential roles of the herb pair HZ and GC in this formula, in the current study, genome-wide microarrays based on thyroid tissues obtained from normal rats, goiter rat model, goiter rats treated with HYD or HYD deleted HZ/GC herb pair were detected to identify candidate goiter-related genes and HYD regulating genes. Then, the disease genes-drug genes interaction network was constructed, and the topological importance of nodes in the created network were evaluated to screen potential targets of HYD acting on goiter rats. After that, a series of experiments were performed to validation our prediction results (Figure 1). This study provides a promising method to explain the rationality of multi-component drugs such as herbal formulae.

## RESULTS

### Chemical compounds of HYD

Liquid chromatography triple quadrupole-tandem mass spectrometry (LC-QQQ-MS/MS) was performed to identify the chemical compounds of each herb containing in HYD. As shown in Supplementary Figure S1 and Supplementary Table S1, 22 representative chemical compositions: Forsythoside B (1.368 μg/mL), Forsythoside A (236.4 μg/mL), Liquiritin (101.4 μg/mL), Ferulic acid (8.832 μg/mL), Peimine (0.584 μg/mL), Narirutin (297.4 μg/mL), Peiminine (0.3747 μg/mL), Hesperidin (366.5 μg/mL), Isoliquiritoside (4.767 μg/mL), Liquiritigenin (1.190 μg/mL), Apigenin (0.09083 μg/mL), Naringenin (1.438 μg/mL), Glycyrrhizic acid (232.0 μg/mL), Hesperetin (223.0 μg/mL), Isoliquiritigenin (0.1687 μg/mL), Isopimpinellin (0.03317 μg/mL), Bergapten (0.2806 μg/mL), Sinensetin (0.3334 μg/mL), Nobiletin (2.951 μg/mL), Tangeretin (0.7314 μg/mL), 5-Demethylnobiletin (0.1187 μg/mL) and Columbianadin (1.003 μg/mL), in HYD were determined.

### Goiter-related genes are implicated into alterations in hypothalamus-pituitary-thyroid/gonad/growth axes

**Figure 2:**
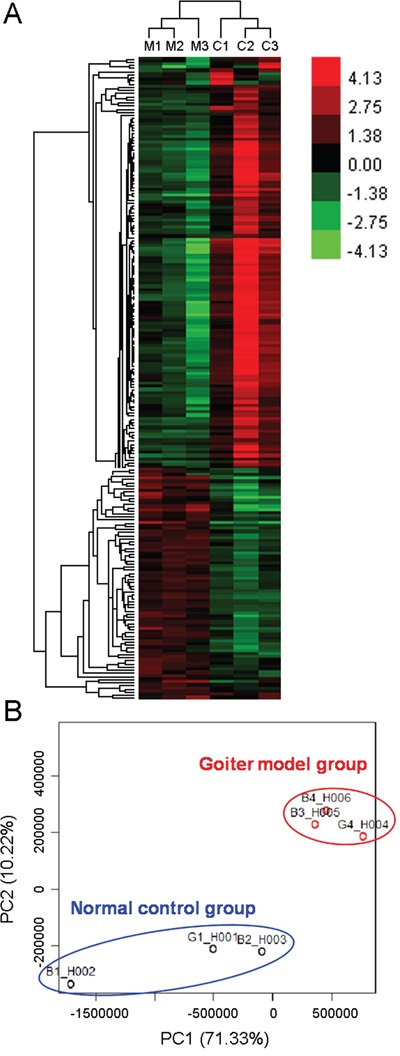
Unsupervised hierarchical clustering analysis (A) and principal component analysis (B) of all dysregulated genes in goiter model and normal control rats. Red represents a high expression while green represents a low expression. M1~M3, three samples in goiter model group; C1~C3, three samples in normal control group

Compared to the normal rats, the serum levels of T3 and T4 were both significantly decreased, while the serum levels of TSH were dramatically increased in rats with oral administration of propylthiouracil for 14 days (Supplementary Figure S2), suggesting the successful construction of goiter rat model caused by iodine deficiency. After data processing and differentially expressed gene (DEG) screening, there were 295 upregulated and 391 downregulated genes in thyroid tissues of goiter rats compared to the normal rats (Supplementary Table S2). In addition, unsupervised hierarchical clustering analysis (Figure 2A) and principal component analysis (Figure 2B) of all dysregulated genes showed a good differentiation of normal and goiter samples.

**Figure 3:**
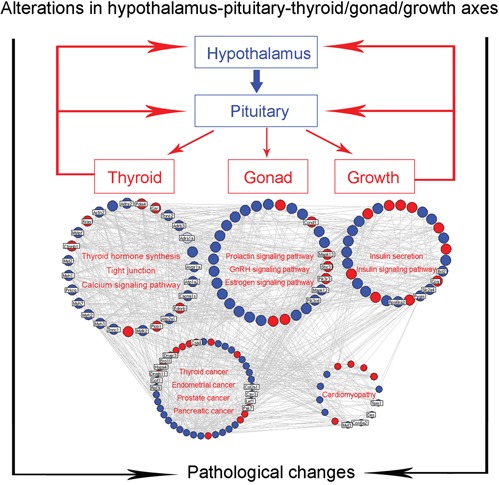
Goiter-related genes are implicated into alterations in hypothalamus-pituitary-thyroid/gonad/growth axes Blue nodes refer to the downregulated genes in goiter model group compared to normal control group; Red nodes refer to the upregulated genes in goiter model group compared to normal control group.

Then, we constructed the goiter imbalance network using interaction information among goiter deregulated genes. This network consists of 519 nodes and 1684 edges (Supplementary Table S3). A total of 139 hub genes, the degree values of which are more than two fold of the median degree of all nodes in the network [24], were identified (Supplementary Table S4). According to the pathway enrichment analysis based on KEGG data, the hub genes of the goiter imbalance network were frequently involved into thyroid hormone synthesis, tight junction, calcium signaling pathway, prolactin signaling pathway, GnRH signaling pathway, estrogen signaling pathway, insulin secretion and insulin signaling pathway, which are closely correlated with the secretion of thyroid hormone, hebin and insulin, suggesting that the hub goiter deregulated genes might be mainly implicated into the alterations in hypothalamus-pituitary-thyroid/gonad/growth axes, leading to the occurrence and progression of various diseases, such as thyroid, endometrial, prostate and pancreatic cancers, and cardiomyopathy (Figure 3).

### HYD alleviates iodine-deficient goiter via regulating thyroid hormone synthesis

#### Network target prediction based on microarray gene expression profiling

Compared to the goiter model rats, the serum levels of T3 and T4 were both significantly increased, while the serum levels of TSH were dramatically decreased in goiter rats treated with HYD for 28 days (Supplementary Figure S3). The DEG screening identified 426 HYD regulating genes: 287 upregulated and 139 downregulated genes in thyroid tissues of goiter rats treated with HYD compared to the goiter model rats (Supplementary Table S5).

Based on our previous developed target prediction system [25], a total of 142 genes were predicted as putative targets of HYD. As shown in Supplementary Table S6, the amount of putative targets hit by HZ, KB, FBX, ZBM, QP, CP, DG, CX, DH, LQ and GC were 11, 3, 1, 5, 48, 25, 30, 25, 30, 53, 37, 30 and 28, respectively.

To shed light on the pharmacological mechanisms of HYD acting on goiter, we constructed disease-related genes-drug-regulating genes network based on the interactions among 139 hub goiter deregulated genes, 10 known goiter related genes, 426 HYD regulating genes and 143 putative targets of HYD. This network consists of 422 nodes and 2039 edges (Supplementary Table S7).

A total of 233 hub nodes, the degree values of which are more than two fold of the median degree of all nodes in the disease-related genes-drug-regulating genes network [24], were identified. Then, the network of hub nodes consists of 233 nodes and 1729 edges (Supplementary Table S8). In addition, 4 topological features, 'Degree,' 'Betweenness', 'Closeness' and 'K coreness' were calculated to identify major hub nodes. As a result, 77 major hub nodes, the ‘Degree', 'Betweenness', 'Closeness' and 'K coreness' of which were all larger than the corresponding median values, were identified as candidate targets of HYD acting on goiter. Please see detailed information on topological features of these candidate targets of HYD acting on goiter in Supplementary Table S9.

**Figure 4:**
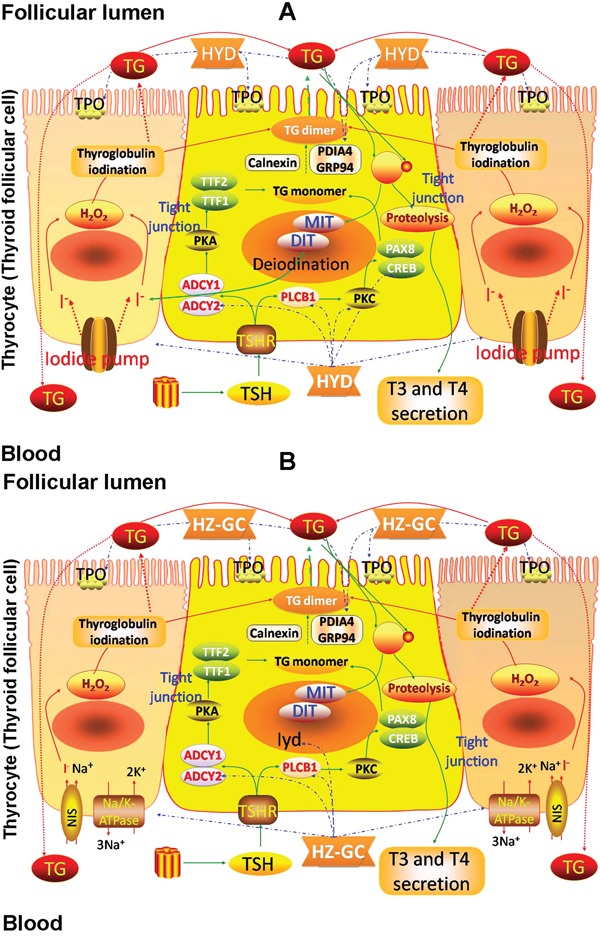
Involvement of HYD (A) and the herb pair HZ-GC (B) in thyroid hormone synthesis via regulating the corresponding candidate targets

According to the pathway enrichment analysis, the candidate targets of HYD acting on goiter were significantly associated with thyroid hormone synthesis, in which there were nine HYD candidate targets involved, including Adcy1 (Adenylate cyclase 1, a putative target of HYD), Adcy2 (Adenylate cyclase 2, downregulated in goiter model rats, but upregulated by HYD treatment), Creb1 (cAMP responsive element binding protein 1, a putative target of HYD), Hspa5 (Heat shock protein 5, a putative target of HYD), Pdia4 (Protein disulfide isomerase family A, member 4, upregulated in goiter model rats, but downregulated by HYD treatment), Plcb1 (Phospholipase C, beta 1, upregulated in goiter model rats, but downregulated by HYD treatment), Prkca (Protein kinase C, alpha, a putative target of HYD), Prkcb (Protein kinase C, beta, a putative target of HYD) and Tpo (thyroid peroxidase, a known therapeutic target of goiter) (Figure 4A), implying that HYD might exert its therapeutic effects on iodine-deficient goiter via regulating thyroid hormone synthesis.

#### Experimental validation based on independent samples

**Figure 5:**
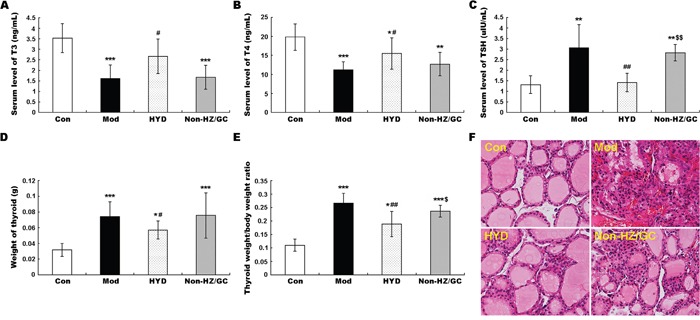
Effect of HYD and the herb pair HZ-GC on severity of iodine-deficient goiter based on propylthiouracil-induced goiter rats **(A)** Reduced levels of T3 in sera of goiter rats could be efficiently increased by the administration of HYD at 0.9 g/(kg·day) for 28 days; **(B)** Reduced levels of T4 in sera of goiter rats could be efficiently increased by the administration of HYD at 0.9 g/(kg·day) for 28 days; **(C)** Elevated serum levels of TSH in goiter rats were significantly decreased in HYD treatment group; **(D)** and **(E)** Weight of thyroid tissues and TBR were respectively increased in the in goiter model group compared to the normal group; After the treatment of HYD, the two parameters were both significantly lower than those in goiter model rats; **(F)** Histological observations of the thyroid tissues in different groups (H&E staining). Data are represented as the mean ± S.E. ‘*’, ‘**’, and ‘***’, P<0.05, P<0.01, and P<0.001, respectively, comparison with the normal control group. '^#^' and '^##^', P<0.05 and P<0.01, respectively, comparison with the model group. '^$^' and '^$$^', P<0.05 and P<0.01, respectively, comparison with the HYD group.

**Figure 6:**
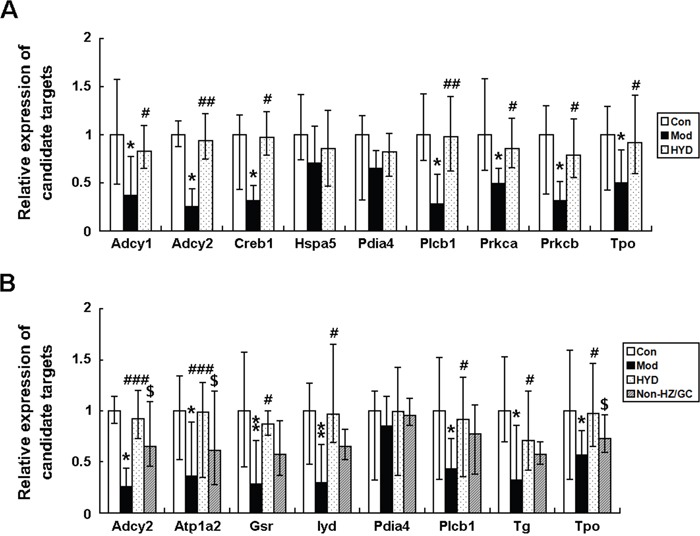
Effect of HYD and the herb pair HZ-GC on mRNA expression levels of the corresponding candidate targets according to real-time PCR analysis Data are represented as the mean ± S.E. ‘*’, ‘**’, and ‘***’, P<0.05, P<0.01, and P<0.001, respectively, comparison with the normal control group. '^#^' and '^##^', P<0.05 and P<0.01, respectively, comparison with the model group. '^$^' and '^$$^', P<0.05 and P<0.01, respectively, comparison with the HYD group.

**Figure 7:**
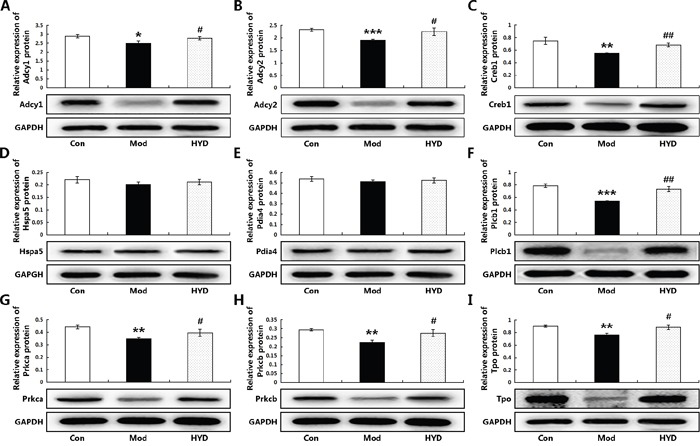
Effect of HYD on protein expression levels of the corresponding candidate targets (A) Adcy1; (B) Adcy2; (C) Creb1; (D) Hspa5; (E) Pdia4; (F) Plcb1; (G) Prkca; (H) Prkcb; (I) Tpo according to Western blot analysis Data are represented as the mean ± S.E. ‘*’, ‘**’, and ‘***’, P<0.05, P<0.01, and P<0.001, respectively, comparison with the normal control group. '^#^' and '^##^', P<0.05 and P<0.01, respectively, comparison with the model group.

To verify this hypothesis, an independent experimental validation was performed. In line with findings based on samples of microarray analysis, the reduced levels of T3 and T4 in sera of goiter rats could be efficiently increased by the administration of HYD at 0.9 g/(kg·day) for 28 days (both P<0.05, Figure 5A and 5B), while the elevated serum levels of TSH in goiter rats were significantly decreased in HYD treatment group (P<0.01, Figure 5C). In addition, the weight of thyroid tissues and the thyroid weight/body weight ratio (TBR) in different groups were also measured at a macroscopic level. As shown in Figure 5D and 5E, there were obvious increases in the weight of thyroid tissues and TBR in goiter model group compared to the normal group (both P<0.001). After the treatment of HYD, the two parameters were both significantly lower than those in goiter model rats (all P<0.05). Moreover, the observations of histological analysis shown in Figure 5F revealed that normal thyroid tissues were characterized of round or oval thyroid follicular cavity, cuboidal follicular epithelial cells and a small amount of blood capillaries around the follicle, which were changed into hyperplasia and hypertrophy of parenchyma, collapsed follicles, scanty colloid contained as well as serious mononuclear cell infiltration in thyroid tissues of goiter model group. Following HYD treatment, the number of follicular cells and height of epithelial cells were markedly increased, and the severity of mononuclear cell infiltration and the blood capillaries reduction were efficiently relieved. More importantly, the real-time PCR analysis based on independent samples showed that the mRNA levels of Adcy1 (P<0.05), Adcy2 (P<0.05), Creb1 (P<0.05), Hspa5, Pdia4, Plcb1 (P<0.05), Prkca (P<0.05), Prkcb (P<0.05) and Tpo (P<0.05) expression in thyroid tissues of goiter rats were all lower than those in normal rats, while the administration of HYD could markedly enhance their expression (except for Hspa5 and Pdia4 genes, all the other genes had differences with statistical significance: all P<0.05, Figure 6A), which were all consistent with the findings of Western blot analysis (except for Hspa5 and Pdia4 proteins, all the other proteins had differences with statistical significance: all P<0.05, Figure 7).

## Herb pair HZ and GC functions as a crucial component in HYD acting on iodine-deficient goiter

### Network target prediction based on microarray gene expression profiling

As shown in Supplementary Figure S3, the reversing effects of HYD without HZ or GC on the dysregulation of serum T3, T4 and TSH levels, and the enlarged thyroid tissues of goiter rats were markedly weaker than those of HYD treatment. Following the microarray data processing and DEG screening, 115 dysregulated genes (46 upregulated and 69 downregulated genes) and 282 dysregulated genes (99 upregulated and 183 downregulated genes) were respectively identified in non-HZ and non-GC groups. Among them, 56 dysregulated genes, commonly identified in both non-HZ and non-GC groups, were defined as HZ-GC-regulating genes (Supplementary Table S10).

To further clarify pharmacological mechanisms of herb pair HZ and GC acting on goiter, we constructed goiter-related genes-HZ-GC-regulating genes network based on the interactions among 139 hub goiter deregulated genes, 10 known goiter related genes, 56 HZ-GC-regulating genes and 28 putative targets of HZ and GC. This network consists of 181 nodes and 629 edges (Supplementary Table S11). According to the pathway enrichment analysis, the 181 nodes were most significantly associated with thyroid hormone synthesis. There are 8 genes, including Adcy2 (downregulated in goiter model rats, but upregulated by HYD treatment and not reversed by HYD without HZ or GC treatment), Atp1a2 (ATPase, Na+/K+ transporting, alpha 2 polypeptide; downregulated in goiter model rats, but upregulated by HYD treatment and not reversed by HYD without HZ or GC treatment), Gsr (glutathione reductase; upregulated in goiter model rats, but not reversed by any drug treatments), Iyd (iodotyrosine deiodinase; a known therapeutic target of goiter), Pdia4 (upregulated in goiter model rats, but downregulated by HYD treatment and not reversed by HYD without HZ or GC treatment), Plcb1 (upregulated in goiter model rats, but downregulated by HYD treatment and not reversed by HYD without HZ or GC treatment), Tg (thyroglobulin, a known therapeutic target of goiter) and Tpo (a known therapeutic target of goiter), involved into thyroid hormone synthesis (Figure 4B). Since the above data showed that HYD treatment could reverse the imbalance in thyroid hormone synthesis during the progression of iodine-deficient goiter (Figures 4–6), and the HZ-GC-regulating genes were also significantly associated with this pathway via interactions with goiter-related genes, we hypothesized that the herb pair HZ and GC might play a crucial role in the therapeutic effects of HYD acting on this disease.

### Experimental validation based on independent samples

**Figure 8:**
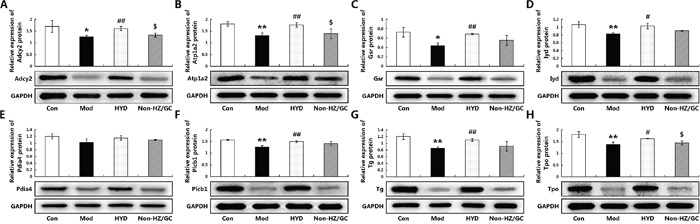
Effect of herb pair HZ-GC on protein expression levels of the corresponding candidate targets (A) Adcy2; (B) Atp1a2; (C) Gsr; (D) Iyd; (E) Pdia4; (F) Plcb1; (G) Tg; (H) Tpo according to Western blot analysis Data are represented as the mean ± S.E. ‘*’, ‘**’, and ‘***’, P<0.05, P<0.01, and P<0.001, respectively, comparison with the normal control group. '^#^' and '^##^', P<0.05 and P<0.01, respectively, comparison with the model group. '^$^' and '^$$^', P<0.05 and P<0.01, respectively, comparison with the HYD group.

The further independent experimental validation showed that the therapeutic effects of HYD on goiter rats were dramatically reduced when the herb pair HZ-GC was deleted from this formula (Figure 5), which was consistent with the findings based on samples of microarray analysis. Moreover, the results of real-time PCR and western blot analyses indicated that both the mRNA and protein levels of Adcy2 (P<0.05), Atp1a2 (P<0.05), Gsr (P<0.05), Iyd (P<0.05), Pdia4, Plcb1 (P<0.05), Tg (P<0.05) and Tpo (P<0.05) expression in thyroid tissues of goiter rats were all lower than those in normal rats, but could be effectively increased by HYD treatment. Notably, when the herb pair HZ-GC was deleted from HYD, its reversing effects on these genes were markedly decreased (Figure 6B and Figure 8).

## DISCUSSION

Owing to the complexity of Chinese herbal formulae, their actions and underlying mechanisms remain unclear. In the current study, we combined the genome-wide microarray detection based on thyroid tissues of goiter rats, the prediction of the target profiles of all available chemical compounds in HYD, and network target analysis in order to define HYD from a systems perspective and at a molecular level. At first, a list of differentially expressed genes with great topological importance in the goiter-related gene interaction network were identified and were mainly involved into the alterations in hypothalamus-pituitary-thyroid/gonad/growth axes. Then, the network target analysis illustrated the links between HYD regulating genes and goiter-related genes, and identified the candidate targets of HYD acting on goiter. Functionally, these candidate targets were most significantly associated with thyroid hormone synthesis. Moreover, the potential regulating genes of the herb pair HZ and GC were also found to function as crucial components in the pathway of thyroid hormone synthesis. These prediction findings were all verified by the experiments based on goiter rats.

Thyroid hormones, one of mammalian hormones with halogen and iodide in the biochemical structure, play vital roles in several biological processes, such as metabolism of carbohydrates, lipids and proteins, and cell replication, in developing and adult mammals [26, 27]. During the synthesis of thyroid hormones, TSH from the pituitary gland couples to Gq/Gs, leading to the activation of PLC and ADCY1/2; Simultaneously, Iodide, functions as the rate-limiting substrate for thyroid hormone biosynthesis, is actively transported into thyroid follicular cells by the sodium-iodide symporter at the basolateral membrane; TPO oxides iodide on the luminal side of the apical membrane, and then iodinates tyrosyl residues of the intrafollicular TG; Subsequently, T4 or T3 are formed by coupling two iodotyrosines, mono- and diiodotyrosyl (MIT, DIT) together; This coupling reaction is catalyzed by TPO and requires the presence of hydrogen peroxide (H_2_O_2_); After that, TG, carrying T4 and T3, is internalized into the follicular cell by micro- and macropinocytosis and digested in lysosomes; The thyronines T4 and T3 are released into the blood stream, while MIT and DIT are deiodinated by a dehalogenase and recycled [28–30]. This complex process is controlled by the hypothalamus-pituitary-thyroid/gonad/growth axes, alterations in which may result in the decreased synthesis of thyroid hormone, with consequent increased thyroid weight, goiter, and hypothyroidism [31, 32]. Consistently, our data based on gene expression profiles and network analysis also revealed the associations of hub genes in the goiter imbalance network with several pathways of hypothalamus-pituitary-thyroid/gonad/growth axes.

Since the normal function of the thyroid gland ultimately depends on appropriate iodide intake, it is extremely necessary to supply iodide for thyroid hormonogenesis. Several herbs containing in HYD, such as HZ and KB, have been indicated to be rich in iodine according to the previous chemical identification [33–35]. Accumulating clinical evidence also show the efficient therapeutic effects of this formula on goiter [13, 14]. More interestingly, our study here identified a list of HYD candidate targets using the combination of differential gene screening and network target prediction, and found that several HYD candidate targets may be key elements in thyroid hormone biosynthesis, such as ADCY1/2, PLCB1, CREB1, HSPA5, PDIA4, PRKCA/PRKCB and TPO. Their decreased expression in goiter rats could be markedly reversed by the treatment of HYD according to further real-time PCR and Western blot validations.

According to the Chinese medicinal literature, the herb pair HZ-GC is defined as one of “eighteen antagonistic medicaments”, which means that the two herbs should not be used in the same prescriptions in order to avoid mutually interference. However, this herb pair has been applied in HYD to treat thyroid-related diseases for several decades. To clarify its roles in HYD, we compared the therapeutic effects (T3, T4, TSH, thyroid weight, TBR and histological changes), and the gene expression profiles of goiter rats treated with HYD and HYD deleted HZ ad GC. As a result, although HYD-non-HZ and/or GC treatment had a trend to amolierate the alterations of goiter rats, the differences were not more significant than HYD treatment, suggesting that the therapeutic effects of HYD-non-HZ and/or GC treatment were weaker than HYD treatment. Notably, our network target prediction and experimental validation both confirmed the implications of the herb pair HZ-GC regulating targets into the synthesis of thyroid hormone, highlighting the critical roles of this herb pair in HYD acting on goiter.

In conclusion, this integrative study combining microarray gene expression profiling, network analysis and experimental validations offers the convincing evidence that HYD may alleviate iodine-deficient goiter via regulating thyroid hormone synthesis and explains the necessity of the herb pair HZ and GC in HYD. Our work provides a novel and powerful means to clarify the mechanisms of action for multi-component drugs such as herbal formulae in a holistic way, which may improve drug development and applications.

## MATERIALS AND METHODS

### Data preparation

#### Chemical compounds containing in HYD

Chemical compounds containing in HYD were obtained from TCM Database@Taiwan [36] (http://tcm.cmu.edu.tw/, Updated in 2012-06-28). The detailed information on the Chemical compounds containing in HYD is described in Supplementary Table S12.

#### Known goiter-related genes

Known goiter-related genes were obtained from the following two databases: (1) DrugBank database [37] (http://www.drugbank.ca/, version: 4.3). We only used those drug-target interactions whose drugs are FDA approved for the treatment of goiter and whose targets are human genes/proteins. Totally, we obtained three known therapeutic targets of goiter (TPO, THRA and THRB). (2) Kyoto Encyclopedia of Genes and Genomes (KEGG) Disease Database [38] (http://www.genome.jp/kegg/, Last updated: October 16, 2012). Totally, we obtained eight known goiter-related genes (KEGG ID: H00251; DUOX2, DUOXA2, FOXI1, IYD, SLC26A4, SLC5A5, TG and TPO). After deleting redundancy, there were ten known goiter related genes collected in this study.

### Drug target prediction for HYD

The putative targets of HYD's chemical compounds were predicted based on the following hypothesis: drugs with similar chemical structure usually bind functionally related targets and exert similar therapeutic effects. This prediction method achieves good prediction performance according to the evaluation in our previous study [25]. Detailed description on this drug target prediction method is provided in Supplementary File Section 1.

### Ethics comments

The current study was approved by the Research Ethics Committee of Institute of Chinese Materia Medica, China Academy of Chinese Medical Sciences, Beijing, China. All procedures have been carried out in accordance with the care and the use of animals of the Center for Laboratory Animal Care, China Academy of Chinese Medical Sciences.

### Animals

A total of 47 Wistar rats (number of male rats=23; number of female rats=24; weight 160-180g) were purchased from Charles River laboratories (production license No: SCXK 2012-0001). The rats were divided into two categories: 15 Wistar rats for microarray analysis and 32 Wistar rats for independent experimental validations. All rats were housed in a room with a constant temperature of 24±1°C (mean±SEM) and with a 12-hour light/dark cycle. Rats were allowed free access to food and water.

### Preparation of HYD

All herbs contained in HYD were purchased from Beijing Tongrentang pharmacy. The HYD used in this study was manufactured by Institute of Chinese Materia Medica, China Academy of Chinese Medical Sciences (Beijing, China) using 11 Chinese herbs at a composition of including HZ (5.5g), KB (9g), FBX (9g), LQ (9g), ZBM (9g), QP (9g), DH (9g), DG (9g), CX (9g), CP (9g) and GC (9g). The mixtures of crude herbs were immersed in 10 volumes of distilled water (v/w) for 30 min, followed by boiling for 1 h. Then, the residue was extracted again for 0.5 h with 5 volumes of water (v/w). Two supernates were both filtered through four layers of gauze, and finally pooled and concentrated to a final density of 0.09 g/mL. Meanwhile, HYD without HZ and/or GC (Non-HZ and/or GC) were separately prepared following the same procedures as mentioned above. In addition, the representative chemical compounds of each herb containing in HYD were determined by LC-QQQ-MS/MS.

### Goiter rat model construction and grouping

For microarray analysis, 15 male/female rats were divided into five groups with the equal number of 3: normal control group (con), goiter model group (mod), goiter rats treated with HYD formula (HYD-treatment group), goiter rats treated with HYD formula without HZ (Non-HZ group) and goiter rats treated with HYD formula without GC (Non-GC group). For independent experimental validation, 32 male/female rats were also divided into four groups with the equal number of 8: normal control group (con), goiter model group (mod), goiter rats treated with HYD formula (HYD-treatment group) and goiter rats treated with HYD formula without HZ and GC (Non-HZ-GC group).

Goiter rat model caused by iodine deficiency was constructed by oral administration of propylthiouracil at a dosage of 0.01 g/(kg·day) for 14 days. Drug treatment of goiter was delivered by oral administration for 28 days. The dosage selection for each treated groups were set at 0.9 g/(kg·day) on the basis of clinical dosage for human. Rats in the control and goiter model groups were all administrated by an equal volume of saline.

### Gene expression profiles

Thyroid tissues were collected from rats in four groups, immersed in Trizol (Invitrogen, CA, USA) and frozen in liquid nitrogen immediately for further microarray detection. Fluorescent aRNA targets were prepared from total RNA samples that were pooled from thyroid tissues using Amino Allyl MessageAmp TM II aRNA Amplification Kit (AM1753, Life Technologies, USA) and Cy5 dyes (Amersham Pharmacia, Piscataway, NJ, USA). Then, the fluorescent aRNA targets were hybridized to the Rat OneArrayR v2 (Phalanx Biotech Group, Taiwan; containing 21707 DNA oligonucleotide probes), scanned with an Agilent's High-Resolution C Scanner (Agilent Technologies, CA, USA) and finally analyzed by GenePix 4.1 software (Molecular Devices). The spots with log_2_ ratio ≥ 1 or log_2_ ratio ≤ −1 and P-value < 0.05 are tested for further analysis. The gene expression microarray data of GSE76817 were obtained from the National Center of Biotechnology Information (NCBI) Gene Expression Omnibus (GEO, http://www.ncbi.nlm.nih.gov/geo/).

### DEG screening

Significant DEGs of the control vs. goiter rat model groups, HYD vs. goiter rat model groups, Non-HZ vs. HYD groups, Non-GC vs. HYD groups were identified using the criteria of P value <0.05 and |log_2_ fold change (FC)|>0.5. The hierarchical clustering analysis was performed for the identified DEGs according to the heat map package in R (version 1.0.2, R Core Team, Vienna, Austria). Cluster analysis of the DEGs was performed by Cluster 3.0, based on Euclidean distance.

### Network construction and analysis

The goiter-related-gene interaction network and the goiter-related-gene-HYD-regulating-gene interaction network were constructed based on the public database STRING (Search Tool for Known and Predicted Protein-Protein Interactions, version 10.0, http://string-db.org/) [39] with the median value of combined scores of all interactions as a threshold. Then, Navigator software (Version 2.2.1) was applied to visualize the interaction network.

Four topological features, node's degree, betweenness, closeness and k-coreness, were calculated to evaluate the importance of a node in the interaction network. The definitions of these network topological features are described in Supplementary File Section 2.

### Pathway enrichment analysis

Pathway enrichment analysis was performed using pathway data obtained from the FTP service of KEGG [22] (http://www.genome.jp/kegg/, Last updated: Oct 16, 2012).

### Biochemical analysis

At the end of the treatments before sacrifice, animals were anesthetized by chloral hydrate (10%, w/v). Blood samples were collected from the abdominal artery and immediately centrifuged at a speed of 3000 r/min for 15 min at 4°C to obtain serum, which were stored at −80°C for biochemical analysis. The serum levels of T3, T4 and TSH in different groups were determined by enzyme-linked immunosorbent assay (ELISA) using the corresponding commercially available kits according to the protocol from manufactures (Rapidbio, CA, USA). All experiments were done in triple.

### Histopathology

After the tissue collection, the thyroid tissues were divided into two categories. One was fixed in 4% paraformaldehyde and embedded in paraffin. Then, 4 μm-thick tissue sections were prepared and stained with hematoxylin and eosin (H&E) for histological studies. H&E stained areas were viewed by the application of an optical microscope (Leica MZ16FA Stereo Microscope, German) to assess the pathologic alterations of thyroid tissues in different groups and to monitor the therapeutic effects of the treatment. All sections were evaluated by two observers who were blinded to the experimental conditions of the animals. The other part was immediately stored in liquid nitrogen at −80°C for real-time PCR analysis.

### Real-time PCR

Gene expression levels of candidate targets of HYD or the herb pair HZ-GC in thyroid tissues obtained from different groups were detected by quantitative real-time PCR. SYBR Green 1 kit was applied according to the manufacturer's protocol (Roche, Mannheim, Germany) and the house-keeping gene glyceraldehyde-3-phosphate dehydrogenase (GAPDH) was used as a reference gene. Then, 2 μg of total RNA was used for reverse transcription by using ABI High-Capacity cDNA reverse transcription Kits. The primer sequences of candidate target genes were listed in Supplementary Table S13. In addition, the mixtures were diluted 10-fold, and 10 μl reaction volumes were used for data analysis using BIO-RAD CFX Manager Version 3.0 software. PCR reactions for each gene were repeated three times. The expression level of each gene was calculated using the 2^−ΔΔCt^ method [40].

### Western blot analysis

Protein expression levels of candidate targets of HYD or the herb pair HZ-GC in thyroid tissues obtained from different groups were detected by Western blotting analysis as described in our previous study [41]. Antibodies against the following proteins were used: Adcy1 (rabbit polyclonal antibody, dilution 1:200, Abcam, Cambridge, UK), Adcy2 (rabbit polyclonal antibody, dilution 1:200, Abcam, Cambridge, UK), Atp1a2 (rabbit monoclonal antibody, dilution 1:500, Abcam, Cambridge, UK), Creb1 (rabbit monoclonal antibody, dilution 1:500, Abcam, Cambridge, UK), Gsr (rabbit monoclonal antibody, dilution 1:2000, Abcam, Cambridge, UK), Hspa5 (rabbit monoclonal antibody, dilution 1:2000, Abcam, Cambridge, UK), Lyd (rabbit polyclonal antibody, dilution 1:1000, Abcam, Cambridge, UK), Pdia4 (rabbit polyclonal antibody, dilution 1:1000, Abcam, Cambridge, UK), Plcb1 (rabbit monoclonal antibody, dilution 1:1000, Abcam, Cambridge, UK), Prkca (rabbit monoclonal antibody, dilution 1:2000, Abcam, Cambridge, UK), Prkcb (rabbit monoclonal antibody, dilution 1:2000, Abcam, Cambridge, UK), Tg (rabbit monoclonal antibody, dilution 1:40000, Abcam, Cambridge, UK), and Tpo (goat polyclonal antibody, dilution 1:500, RD, Minnesota, US). All experiments were performed in triplicate. The mean normalized protein expression ± S.D. was calculated from three independent experiments.

### Statistical analysis

All statistical analyses were performed by the software of SPSS version 11.0 for Windows (SPSS Inc, Chicago, IL, USA). Our data expressed as means ± standard deviation (SD). All parameters were analyzed by one-way analysis of ANOVA followed by LSD test. *P* values less than 0.05 were considered as statistically significant.

## SUPPLEMENTARY DATA FIGURE AND TABLES


























