# Supplementary material for: Thyroid hormone synthesis: a potential target of a Chinese herbal formula Haizao Yuhu Decoction acting on iodine-deficient goiter
Source: Oncotarget. 2016 Jun 30;7(32):51699–712. doi: 10.18632/oncotarget.10329 (PMC5239508; doi:10.18632/oncotarget.10329)
Supplement: Supplementary file 1 [file oncotarget-07-51699-s001.pdf]

Thyroid hormone synthesis: a potential target of a Chinese herbal formula Haizao Yuhu Decoction acting on iodine-deficient goiter

SUPPLEMENTARY DATA

Section 1: Drug target prediction for HYD

The putative targets of HYD's compositive compounds were predicted based on the following hypothesis: drugs with similar chemical structure usually bind functionally related targets. We used Drug Similarity Search tool in Therapeutic Targets Database [1] (TTD, http://xin.cz3.nus.edu.sg/group/cjttd/ttd.asp, Version 4.3.02 release on Aug 25th 2011) to screen similar drugs of compositive compounds containing in HYD through the structural similarity comparison. We only selected the drugs with high similar score (>0.85, similar ~ very similar) in the comparison with the structures of compositive compounds containing in HYD. The therapeutic targets of these similar drugs were also collected as putative targets of HYD.

Section 2: Defining network topological feature set

For each node i in interaction network, we defined four measures for assessing its topological property: (1) 'Degree' is defined as the number of links to node i; (2) 'Node betweenness' is a measure of how often a node is located on the shortest path between other nodes in the network, and is used to evaluate the degree to which the node under study can function as a point of control in the communication. If a node with a high level of betweenness were to be deleted from a network, the network would fall apart into otherwise coherent clusters. Thus, 'Node betweenness' is defined as the number of shortest paths between pairs of nodes that run through node i. (3)'Closeness'is defined as the inverse of the farness which is the sum of node i distances to all other nodes. The Closeness centrality can be regarded as a measure of how long it will take to spread information from node i to all other nodes sequentially. Degree, node betweenness and closeness centralities can measure a node's topological importance in the network. The larger a node's degree/node betweenness /closeness centrality is, the more important the node is in the interaction network [2]. (4) K-core analysis is an iterative process in which the nodes are removed from the networks in order of least-connected [3]. The core of maximum order is defined as the main core or the highest k-core of the network. A k-core sub-network of the original network can be generated by recursively deleting vertices from the network whose degree is less than k. This results in a series of sub-networks that gradually reveal the globally central region of the original network. On this basis, 'K value' is used to measure the centrality of node i.

REFERENCES

1. Zhu F, Shi Z, Qin C, Tao L, Liu X, Xu F, Zhang L, Song Y, Liu X, Zhang J, Han B, Zhang P, Chen Y. Therapeutic target database update 2012: a resource for facilitating target-oriented drug discovery. Nucleic Acids Res. 2012; 40: D1128- D1136.
2. Wang Y, Liu Z, Li C, Li D, Ouyang Y, Yu J, Guo S, He F, Wang W. Drug target prediction based on the herbs components: the study on the multitargets pharmacological mechanism of qishenkeli acting on the coronary heart disease. Evid Based Complement Alternat Med. 2012; 2012:698531. doi: 10.1155/2012/698531.
3. Wuchty, S., Almaas, E. Evolutionary cores of domain co-occurrence networks. BMC Evol Biol. 2005; 5:24.

1 Forsythoside B (Forsythia suspensa)

Reference

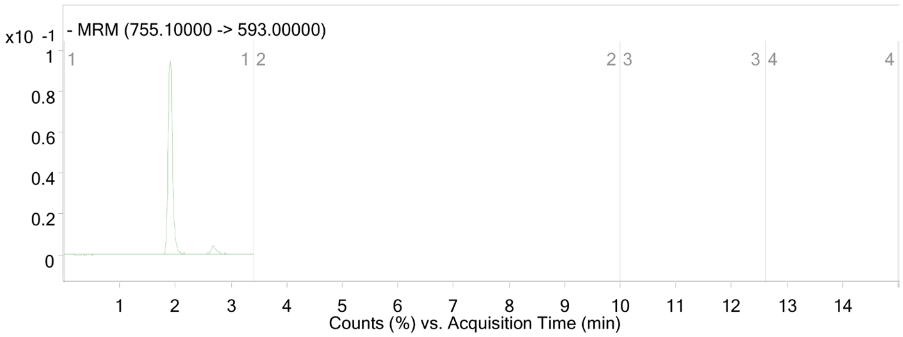

Sample

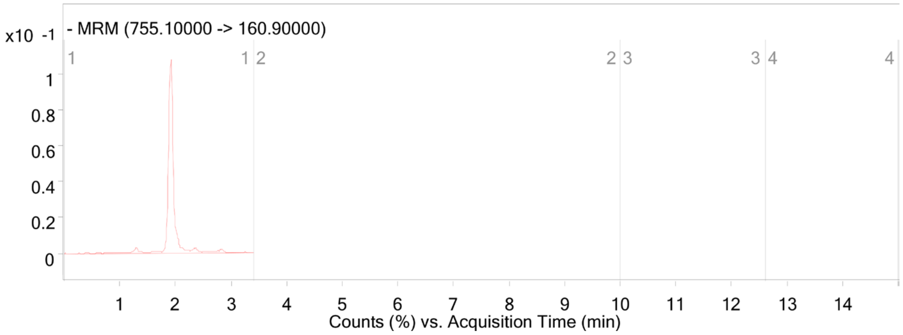

2 Forsythoside A (Forsythia suspensa)

Reference

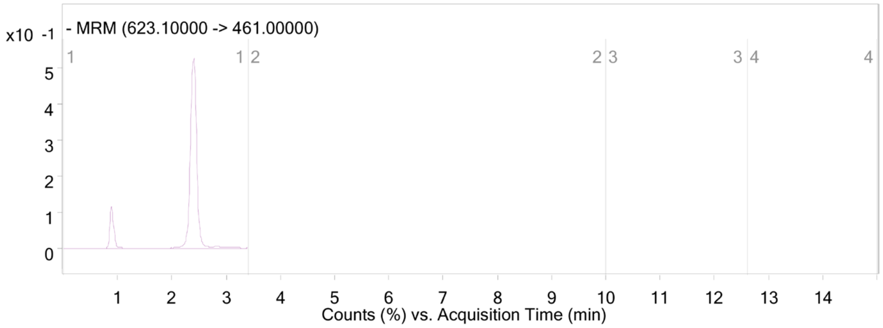

Sample

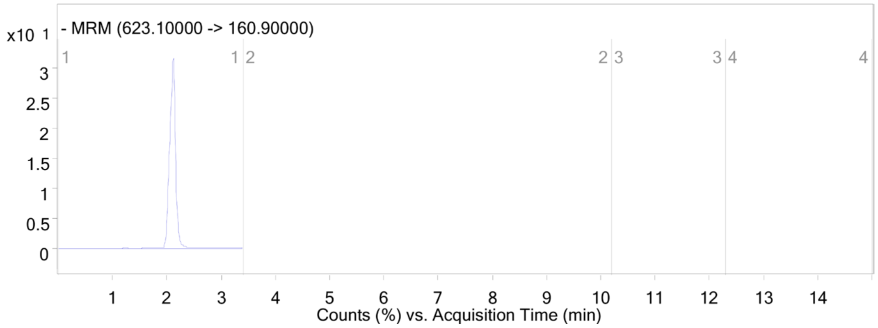

(*Continued*)

3 Liquiritin (Glycyrrhiza uralensis)

Reference

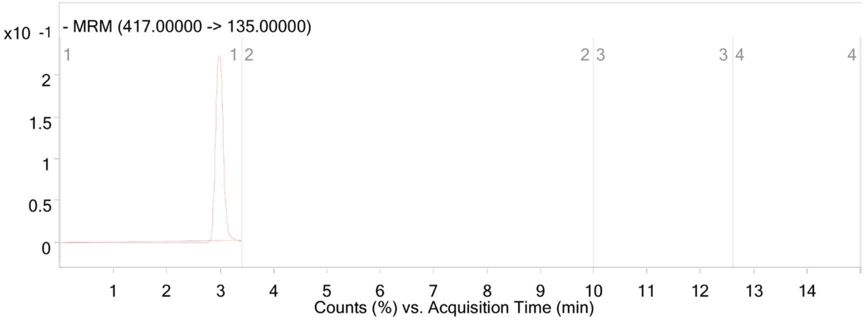

Sample

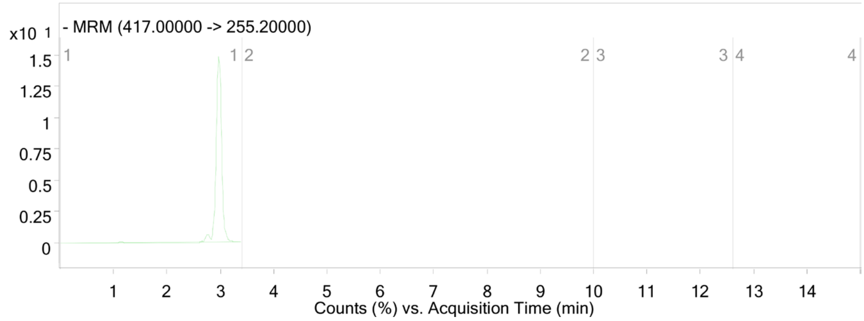

4 Ferulic acid (Angelica sinensis)

Reference

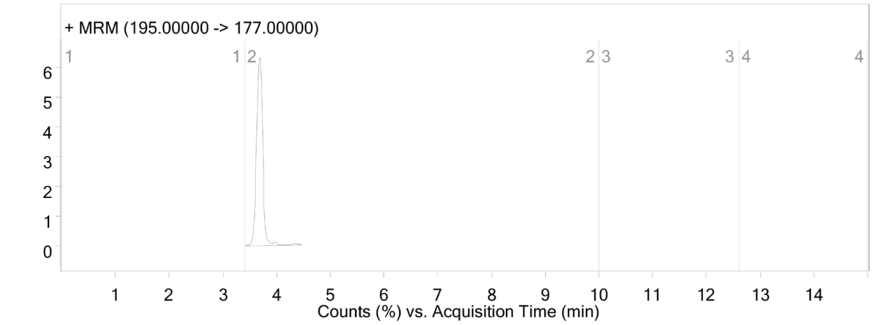

Sample

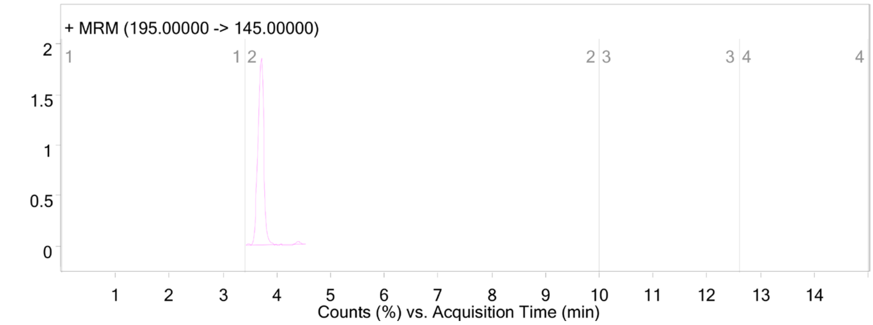

(*Continued*)

5 Peimine (Fritillaria thunbergii)

Reference

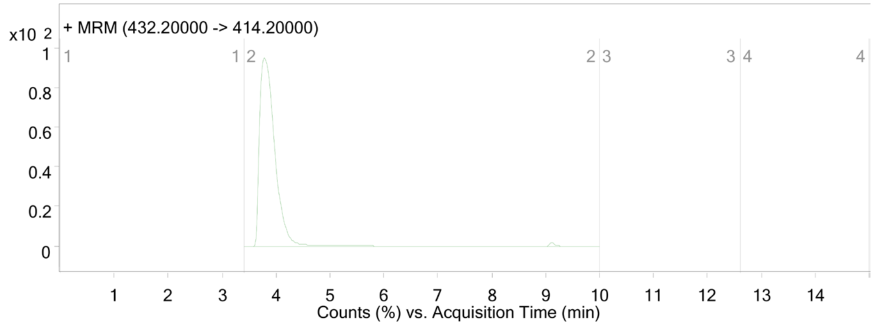

Sample

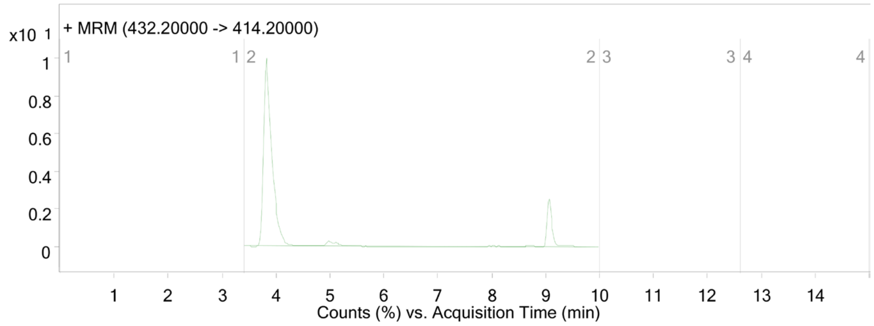

6 Narirutin (Citrus reticulata)

Reference

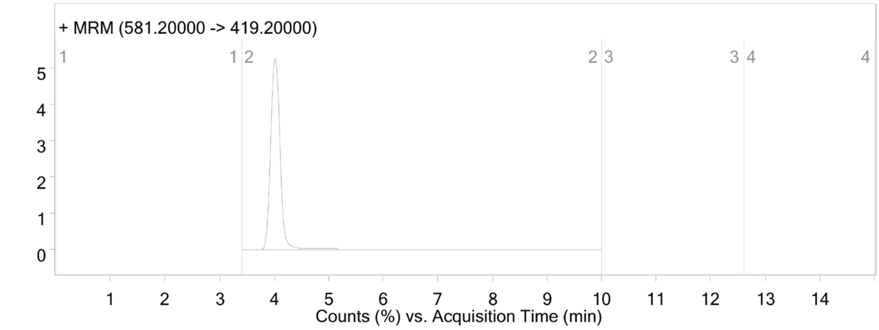

Sample

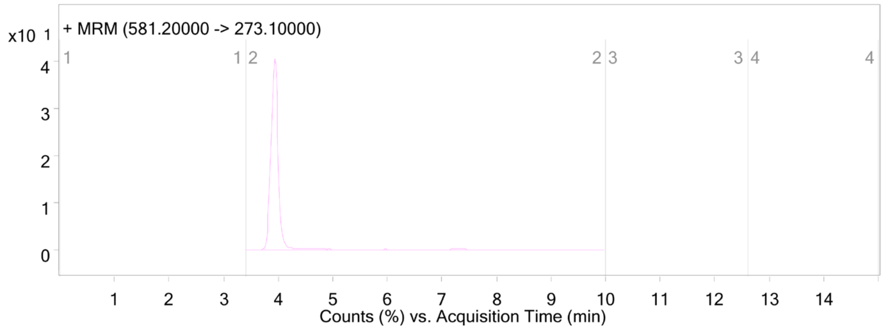

(*Continued*)

7 Peiminine (Fritillaria thunbergii)

Reference

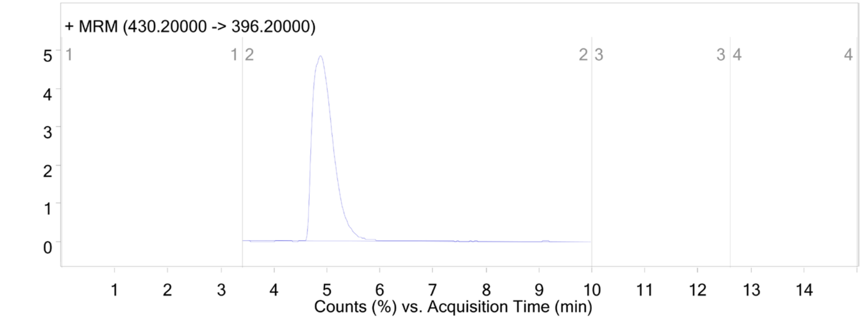

Sample

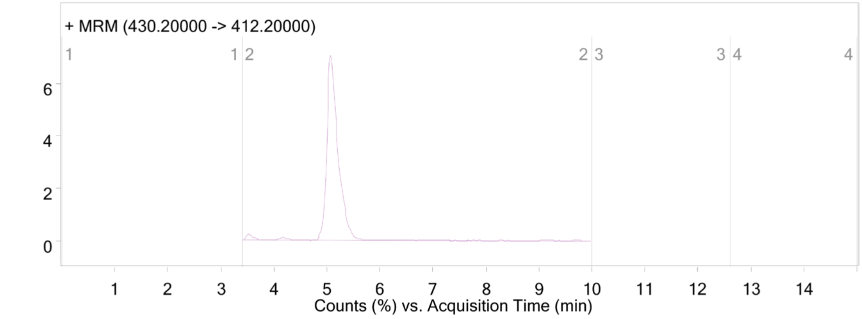

8 hesperidin (Citrus reticulata)

Reference

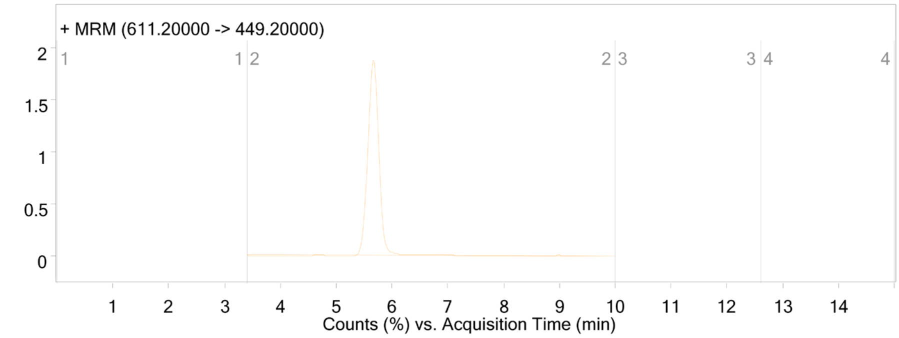

Sample

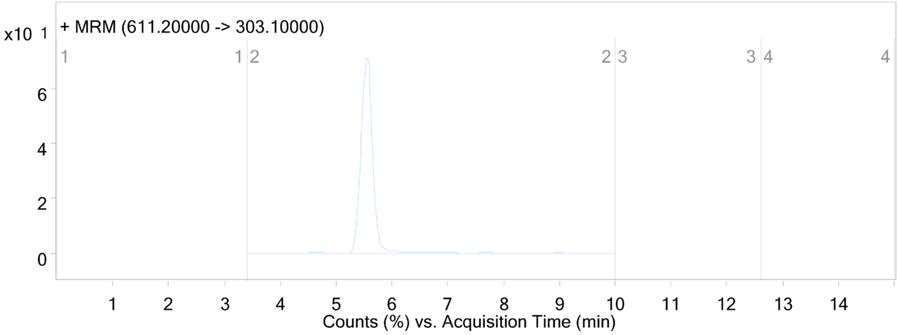

(*Continued*)

9 Isoliquiritoside (Glycyrrhiza uralensis)

Reference

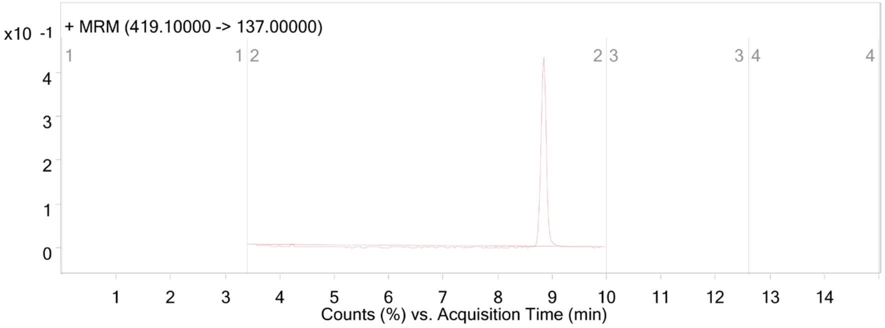

Sample

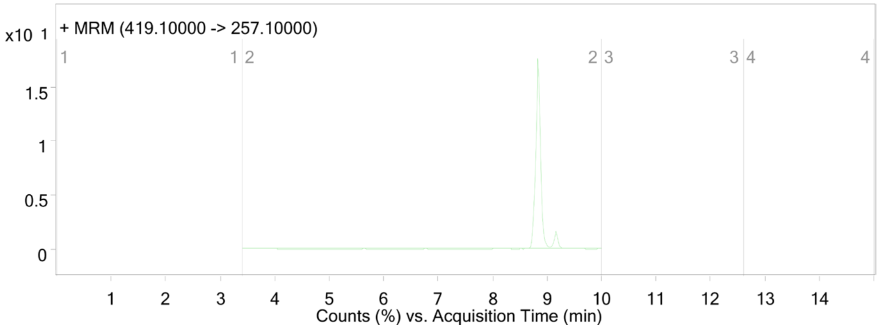

10 Liquiritigenin (Glycyrrhiza uralensis)

Reference

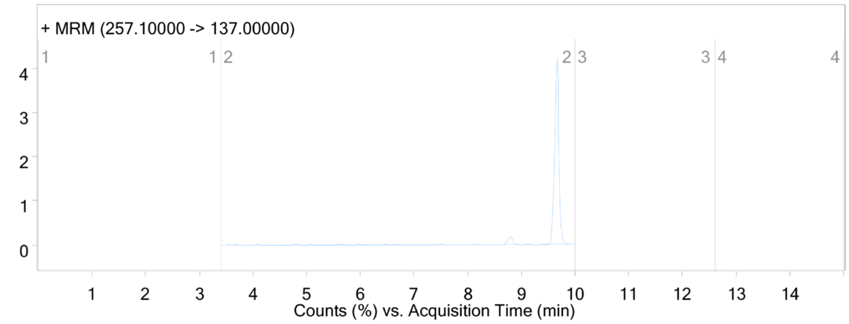

Sample

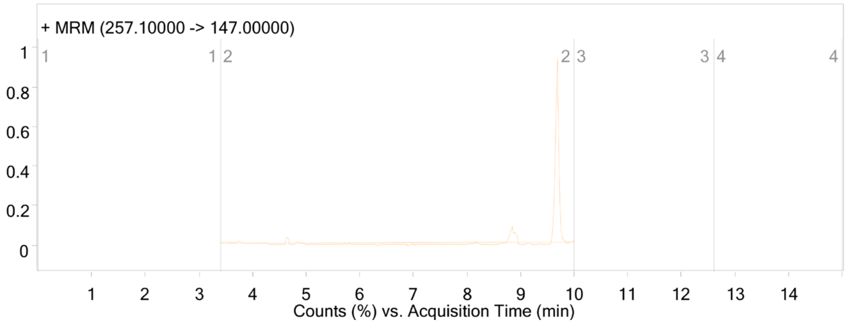

(*Continued*)

11 Apigenin (Citrus reticulata/Ligusticum)

Reference

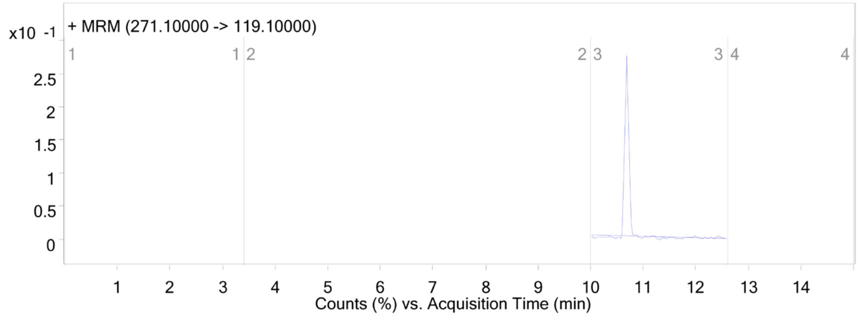

Sample

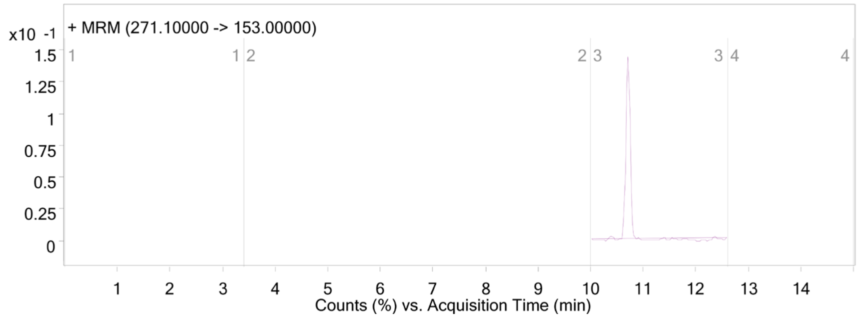

12 Naringenin (Citrus reticulata)

Reference

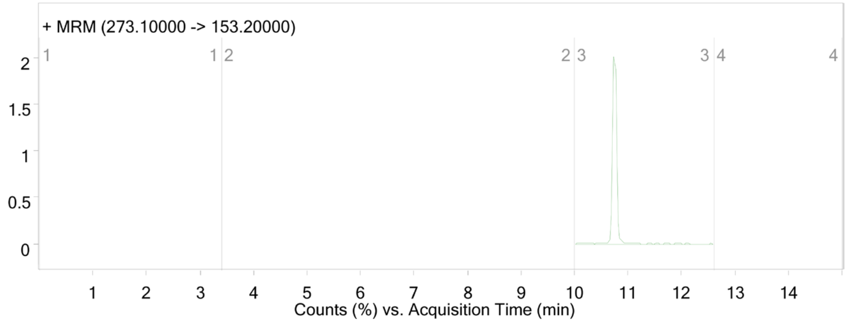

Sample

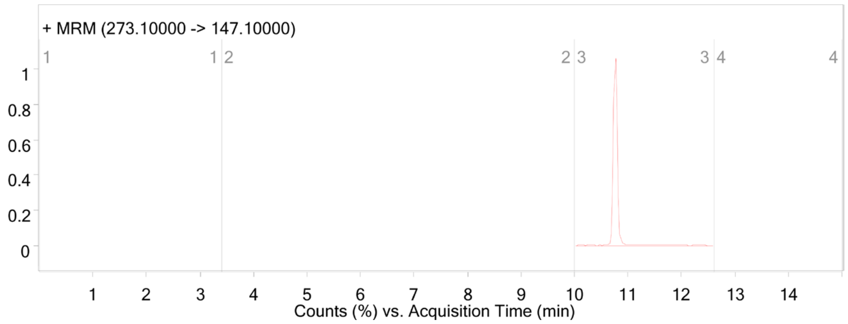

(*Continued*)

13 glycyrrhizic acid (Glycyrrhiza uralensis)

Reference

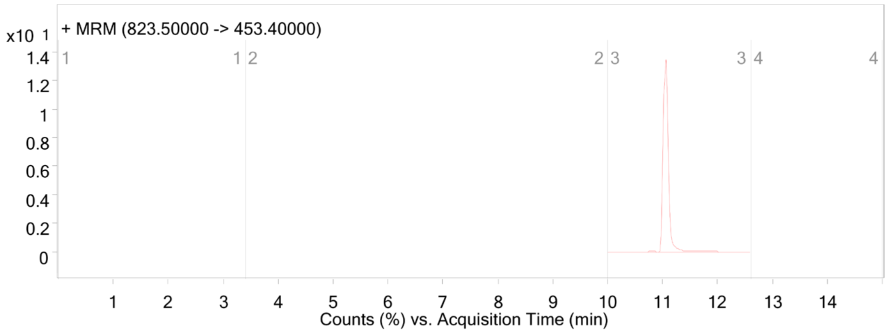

Sample

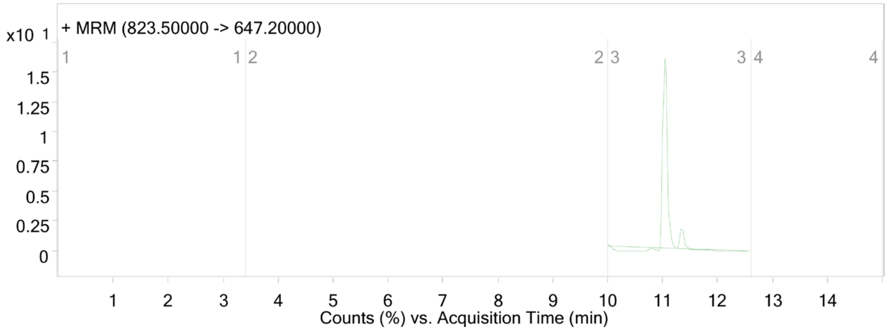

14 Hesperetin (Citrus reticulata)

Reference

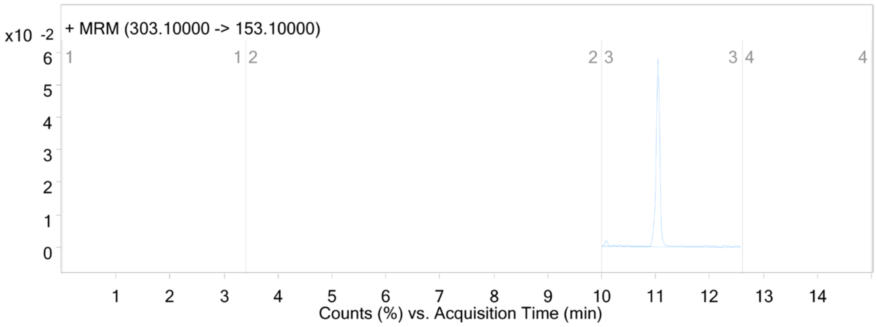

Sample

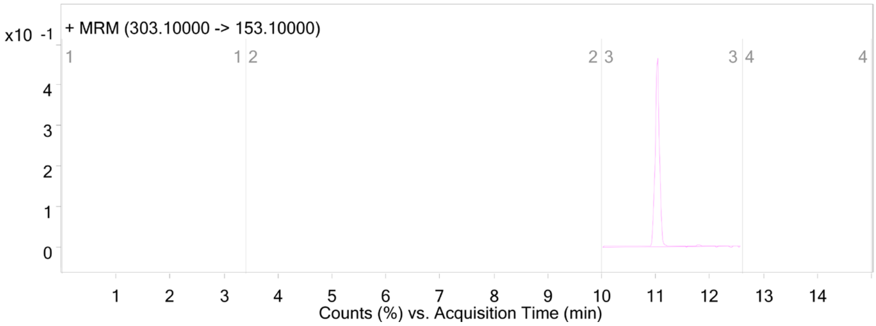

(*Continued*)

15 Isoliquiritigenin (Glycyrrhiza uralensis)

Reference

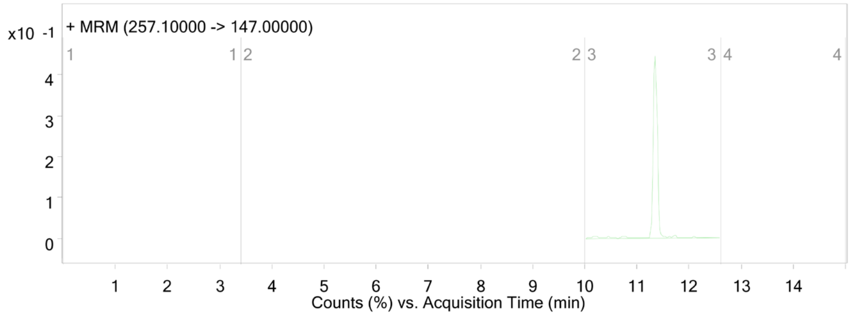

Sample

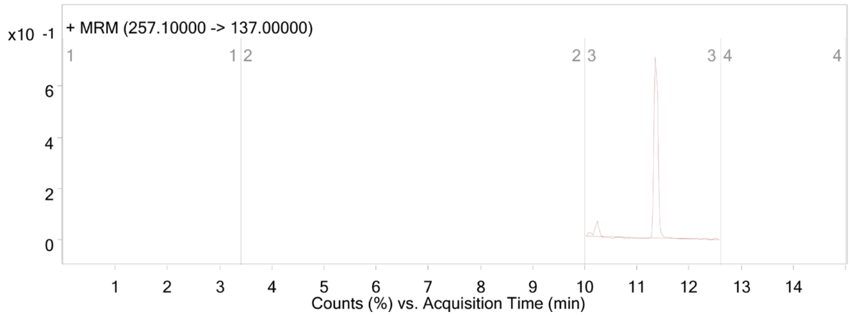

16 Isopimpinellin (Angelica sinensis/Heracleum hemsleyanum)

Reference

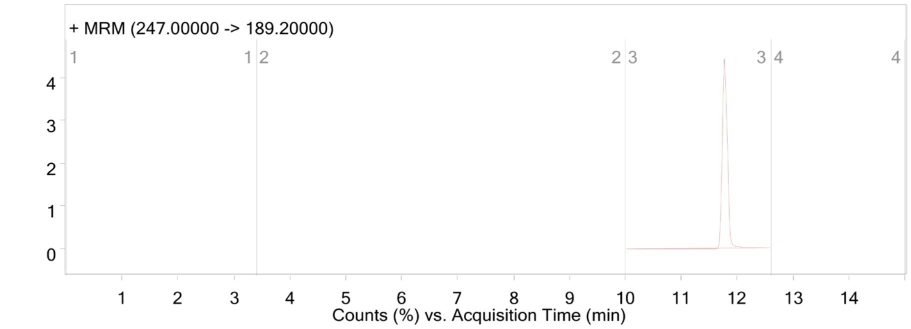

Sample

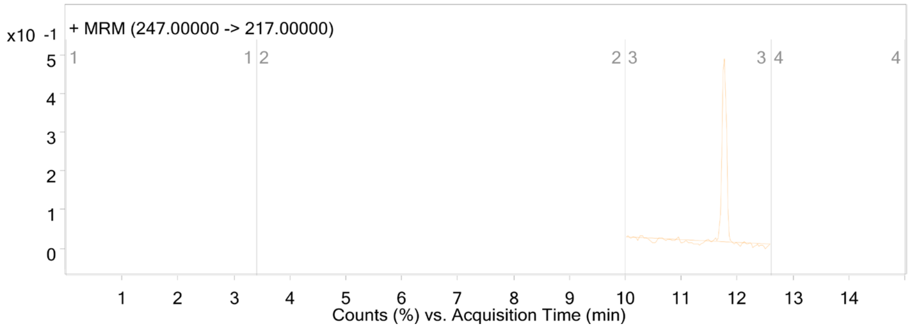

(Continued)

17 Bergapten (Heracleum hemsleyanum)

Reference

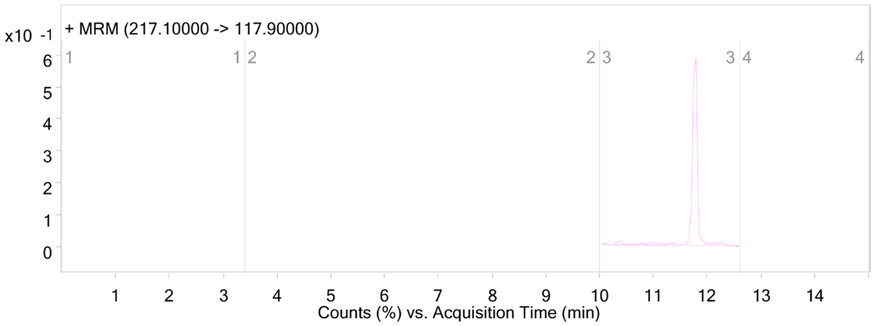

Sample

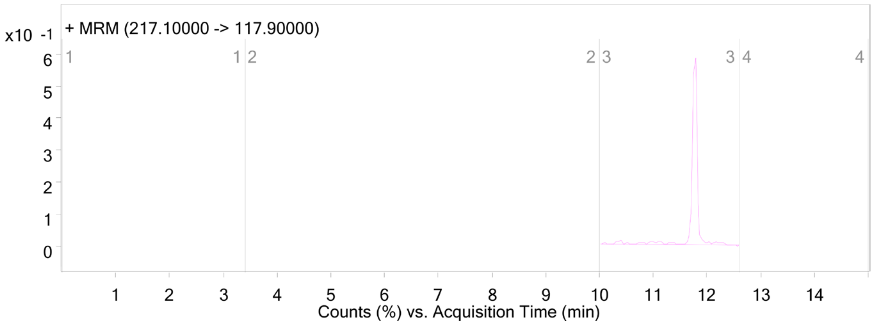

18 sinensetin (Citrus reticulata)

Reference

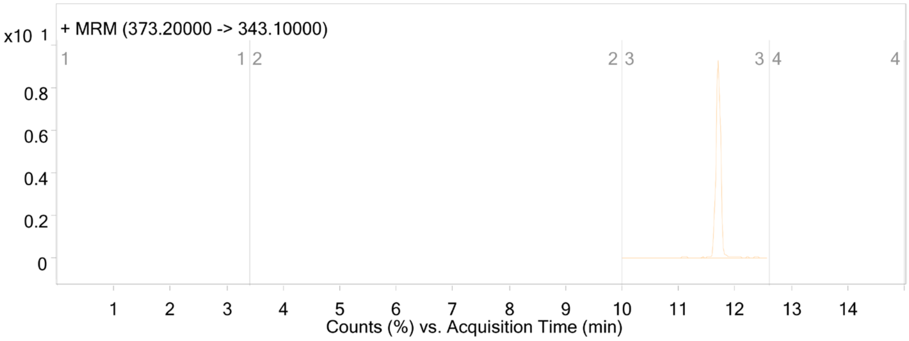

Sample

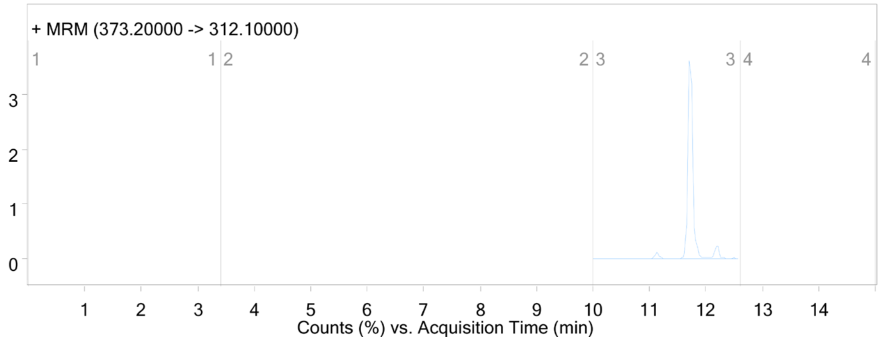

(*Continued*)

19 Nobiletin (Citrus reticulata)

Reference

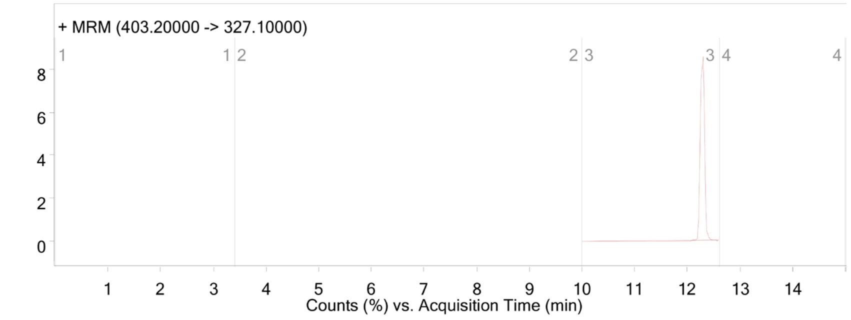

Sample

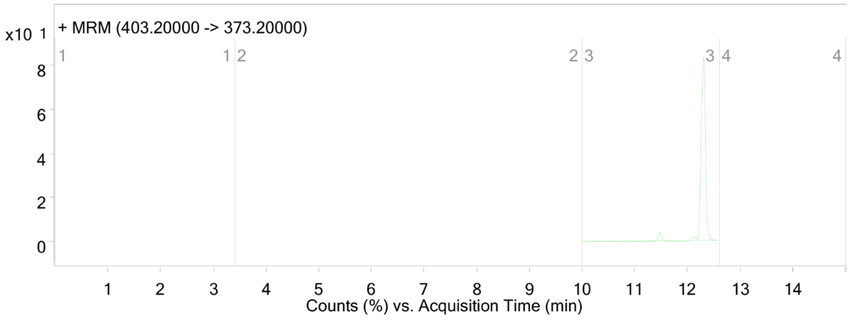

20 Tangeretin (Citrus reticulata)

Reference

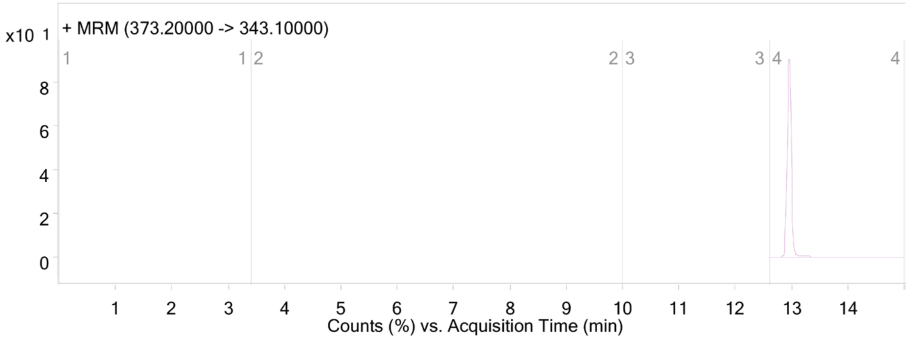

Sample

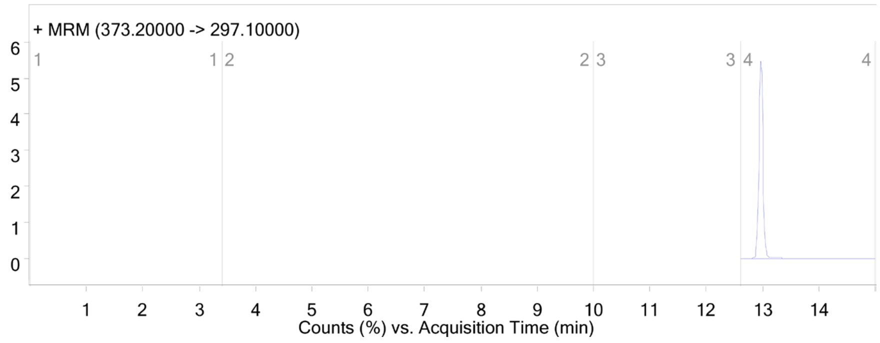

(*Continued*)

21 5-Demethylnobiletin (Citrus reticulata)

Reference

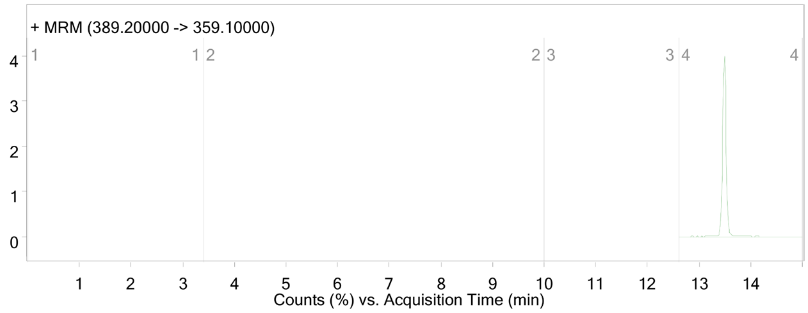

Sample

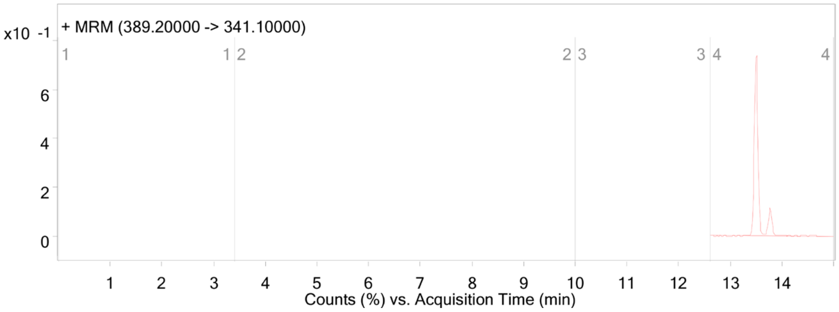

22 Columbianadin (Heracleum hemsleyanum)

Reference

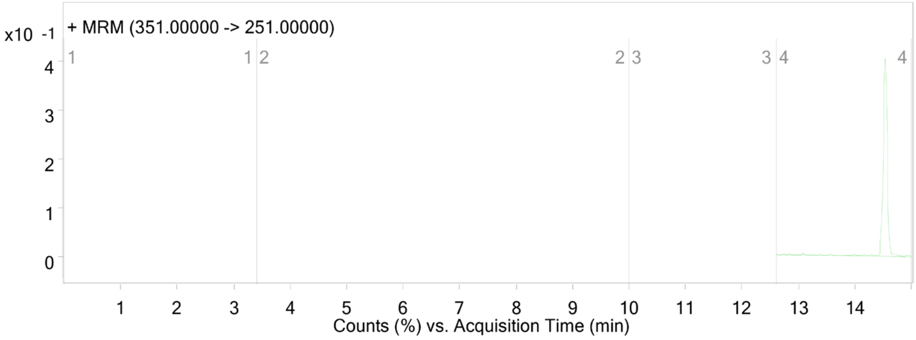

Sample

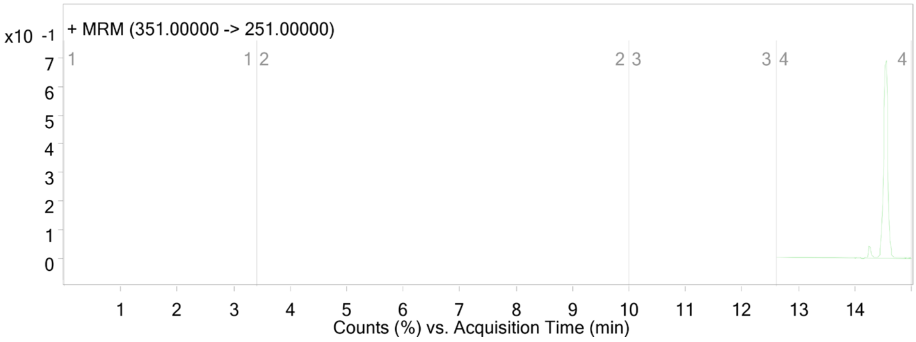

Supplementary Figure S1: Reference and Samples LC–QQQ MS/MS map, which depicts the chemical compounds of each herb containing in HYD.

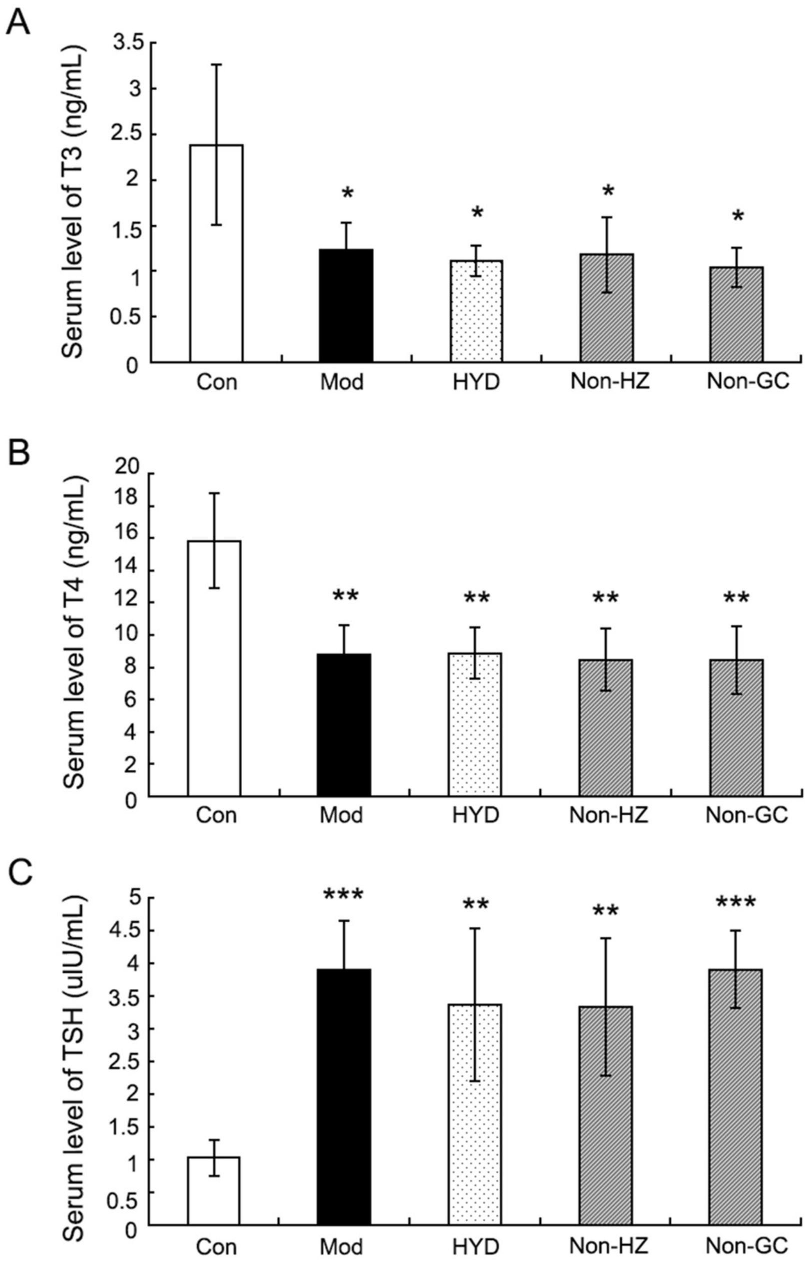

Supplementary Figure S2: Effect of propylthiouracil on thyroid hormones and TSH in wistar rats after oral administrated for 14 days. A. shows the serum levels of T3 in rats on day 14 of propylthiouracil administration. **B.** displays the serum levels of T4 in rats on day 14 of propylthiouracil administration. **C.** shows the serum levels of TSH in rats on day 14 of propylthiouracil administration. Compared to the normal rats, the serum levels of T3 and T4 were both significantly decreased, while the serum levels of TSH were dramatically increased in rats with oral administration of propylthiouracil for 14 days. Data are represented as the mean ± S.E. '*', '**', and '***', P<0.05, P<0.01, and P<0.001, respectively, comparison with the normal control group. '#' and '##', P<0.05 and P<0.01, respectively, comparison with the model group. '$' and '$$', P<0.05 and P<0.01, respectively, comparison with the HYD group.

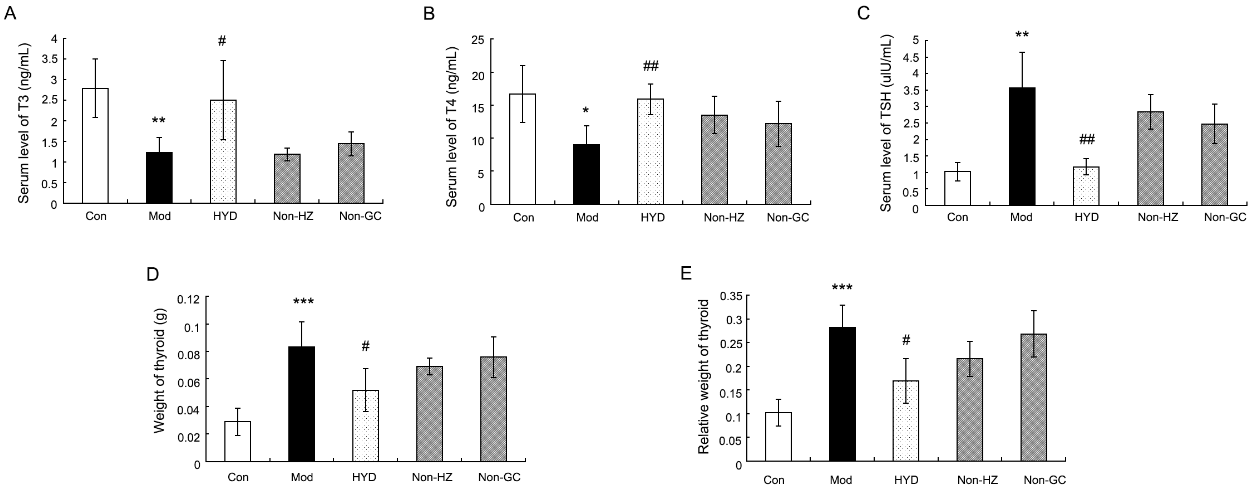

Supplementary Figure S3: Effect of HYD, and HYD deleted HZ or GC on severity of iodine-deficient goiter based on propylthiouracil-induced goiter rats. A. shows the serum levels of T3 in goiter rats after a duration of 28 days' treatment. **B.** displays the serum levels of T4 in goiter rats after a duration of 28 days' treatment. **C.** shows the serum levels of TSH in goiter rats after a duration of 28 days' treatment. **D.** and **E.** show the weight of thyroid and thyroid weight/body weight ratio respectively in goiter rats after a duration of 28 days' treatment. Data are represented as the mean ± S.E. '*', '**', and '***', $P<0.05$, $P<0.01$, and $P<0.001$, respectively, comparison with the normal control group. '#' and '##', $P<0.05$ and $P<0.01$, respectively, comparison with the model group.

Supplementary Table S1: Detailed information of 22 representative chemical compositions in HYD determined by HPLC analysis

See Supplementary File 1

Supplementary Table S2: List of dysregulated genes in goiter rats compared to normal rats. After data processing and DEG screening, there were 295 upregulated and 391 downregulated genes in thyroid tissues of goiter rats compared to the normal rats

See Supplementary File 2

Supplementary Table S3: Interaction information of goiter imbalance network. The goiter imbalance network were constructed using interaction information among goiter deregulated genes, consisting of 519 nodes and 1684 edges

See Supplementary File 3

Supplementary Table S4: List of hub goiter deregulated genes in the goiter imbalance network. There are a total of 139 hub genes, which were identified by the degree values that are more than two fold of the median degree of all nodes in the network

See Supplementary File 4

Supplementary Table S5: List of dysregulated genes in goiter rats treated with HYD compared to goiter model rats. The DEG screening identified 426 HYD regulating genes: 287 upregulated and 139 downregulated genes in thyroid tissues of goiter rats treated with HYD compared to the goiter model rats

See Supplementary File 5

Supplementary Table S6: Detailed information of putative targets of HYD. It has been indicated that the amount of putative targets hit by HZ, KB, FBX, ZBM, QP, CP, DG, CX, DH, LQ and GC were 11, 3, 1, 5, 48, 25, 30, 25, 30, 53, 37, 30 and 28, respectively

See Supplementary File 6

Supplementary Table S7: Detailed information on goiter-related genes-HYD-regulating genes interaction network. The network was constructed based on the interactions among 139 hub goiter deregulated genes, 10 known goiter related genes, 426 HYD regulating genes and 143 putative targets of HYD. This network consists of 422 nodes and 2039 edges.

See Supplementary File 7

Supplementary Table S8: Detailed information of hub genes in goiter-related genes-HYD-regulating gens interaction network. A total of 233 hub nodes, the degree values of which are more than two fold of the median degree of all nodes in the disease-related genes-drug-regulating genes network, were identified. Then, the network of hub nodes consists of 233 nodes and 1729 edges

See Supplementary File 8

Supplementary Table S9: Detailed information on topological features of candidate targets of HYD acting on goiter. Four topological features, 'Degree,' 'Betweenness', 'Closeness' and 'K coreness' were calculated to identify major hub nodes. As a result, 77 major hub nodes, the ‘Degree', 'Betweenness', 'Closeness' and 'K coreness' of which were all larger than the corresponding median values, were identified as candidate targets of HYD acting on goiter.

See Supplementary File 9

Supplementary Table S10: List of HZ-GC-regulating genes. Following the microarray data processing and DEG screening, 115 dysregulated genes (46 upregulated and 69 downregulated genes) and 282 dysregulated genes (99 upregulated and 183 downregulated genes) were respectively identified in non-HZ and non-GC groups. Among them, 56 dysregulated genes, commonly identified in both non-HZ and non-GC groups, were defined as HZ-GC-regulating genes

See Supplementary File 10

Supplementary Table S11: Interaction data of goiter-related genes-HZ-GC-regulating genes network. The network was constructed based on the interactions among 139 hub goiter deregulated genes, 10 known goiter related genes, 56 HZ-GC-regulating genes and 28 putative targets of HZ and GC. This network consists of 181 nodes and 629 edges

See Supplementary File 11

Supplementary Table S12: Detailed information on the chemical compounds containing in HYD.

See Supplementary File 12

Supplementary Table S13: Primer sequences of candidate targets of HYD and the herb pair HZ-GC

Gene Name	Primer name	Primer sequence
Adcy1	Adcy1-F	TGGATGAGATCAACTACCAGTCC
	Adcy1-R	GATCACTCCAGCGACCACG
Creb1	Creb1-F	AAGCTGCCTCTGGTGATGTA
	Creb1-R	AGGACGCCATAACAACTCCA
Hspa5	Hspa5-F	CAGGGAGAGGAGGAATTGGC
	Hspa5-R	ACCAGATGTGCATGACCCAA
Prkca	Prkca -F	TTTGTGATGCTCATGTTTCCAGTC
	Prkca -R	ATAGAGTGCCAGTGTGTGGGG
Adcy2	Adcy2-F	TCTCATTGCCAGTGGTCGTC
	Adcy2-R	GGCTGTGAGGACTAATTCTGGT
Pdia4	Pdia4-F	ACTGTAGTGGACAGCTTTGGC
	Pdia4-R	TCAGTATCAGAGCATGAGGTGG
Plcb1	Plcb1-F	AATTGGCAGTGACCCAAATCTC
	Plcb1-R	TTCGACCCATTTCTTCTCTGTCC
Tpo	Tpo-F	TGCCTCTCCTACACAGTAAGG
	Tpo-R	GTCTGGAAGGATCTGGAGTCT
Prkcb	Prkcb-F	GCTGTAGTTTCTAATACTGTGTGTC
	Prkcb-R	CCTTTAGGACCTTGCTAGAGTG
Atp1a2	Atp1a2-F	TCCCAAAGGCTACTGTCTCATC
	Atp1a2-R	CGGTGTCCCATTTTGACTTCC
Gsr	Gsr-F	CCAGAATACCAATGTCAAAGGCG
	Gsr-R	AGTCTATGGGCGAGTTTCCG
Iyd	Iyd-F	AAGTGTTCTTTCTGCTCTCTGCT
	Iyd-R	AGACTCCCTATGAGAATCAGTGAC
Tg	Tg-F	ACCCATCTCGTGAGGCTTTC
	Tg-R	TGATAGAAGGTCAAGGTGGACTG
GAPDH	GAPDH-F	CATGAGAAGTATGACAACAGCCT
	GAPDH-R	AGTCCTTCCACGATACCAAAGT
