# Supplementary material for: Thyroid hormone synthesis: a potential target of a Chinese herbal formula Haizao Yuhu Decoction acting on iodine-deficient goiter
Source: Oncotarget. 2016 Jun 30;7(32):51699–712. doi: 10.18632/oncotarget.10329 (PMC5239508; doi:10.18632/oncotarget.10329)
Supplement: Supplementary file 2 [file oncotarget-07-51699-s002.docx]

**Table S1 Detailed information of 22 representative chemical compositions in HYD determined by HPLC analysis**

| **Herb name** | **Component name** | **Content**  **(μg/mL)** | **RSD(%)** | **Molecular formula** | **Chemical constitution** |
| --- | --- | --- | --- | --- | --- |
| *Forsythia suspensa* | Forsythoside B | 1.368 | 2.13 | C_34_H_44_O_19_ | 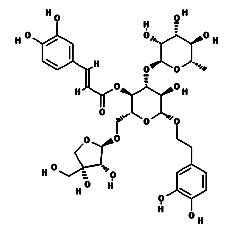 |
| *Forsythia suspensa* | Forsythoside A | 236.4 | 1.69 | C_29_H_36_O_15_ | 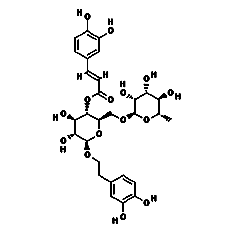 |
| *Glycyrrhiza uralensis* | Liquiritin | 101.4 | 3.12 | C_21_H_22_O_9_ | 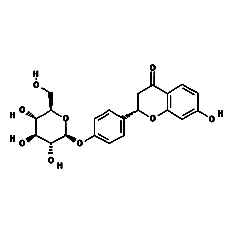 |
| *Angelica sinensis* | Ferulic acid | 8.832 | 0.78 | C_10_H_9_O_4_ | 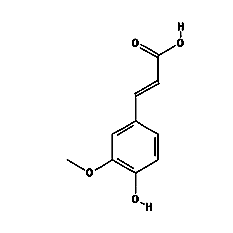 |
| *Fritillaria thunbergii* | Peimine | 0.5840 | 2.22 | C_27_H_45_NO_3_ | 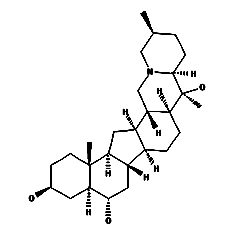 |
| *Citrus reticulata* | Narirutin | 297.4 | 0.98 | C_27_H_32_O_14_ | 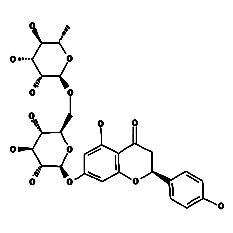 |
| *Fritillaria thunbergii* | Peiminine | 0.3747 | 1.71 | C_27_H_43_NO_3_ | 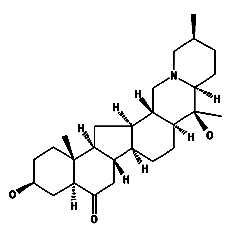 |
| *Citrus reticulata* | Hesperidin | 366.5 | 1.12 | C_28_H_34_O_15_ | 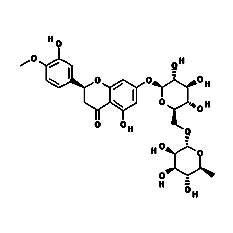 |
| *Glycyrrhiza uralensis* | Isoliquiritoside | 4.767 | 2.74 | C_21_H_22_O_9_ | 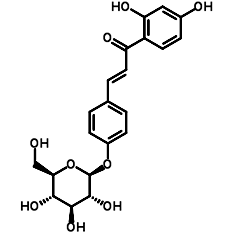 |
| *Glycyrrhiza uralensis* | Liquiritigenin | 1.190 | 0.88 | C_15_H_12_O_4_ | 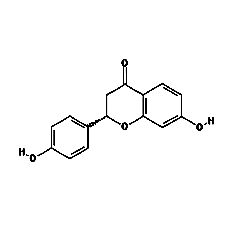 |
| *Citrus reticulata*  *Ligusticum chuanxiong* | Apigenin | 0.09083 | 1.53 | C_15_H_10_O_5_ | 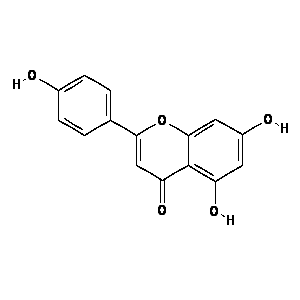 |
| *Citrus reticulata* | Naringenin | 1.438 | 2.73 | C_15_H_12_O_5_ | 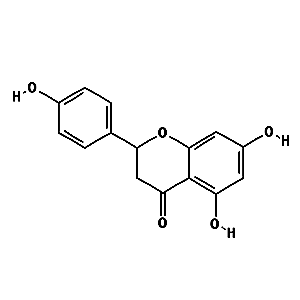 |
| *Glycyrrhiza uralensis* | glycyrrhizic acid | 232.0 | 1.96 | C_42_H_62_O_16_ | 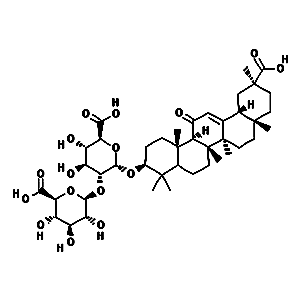 |
| *Citrus reticulata* | Hesperetin | 223.0 | 2.07 | C_16_H_14_O_6_ | 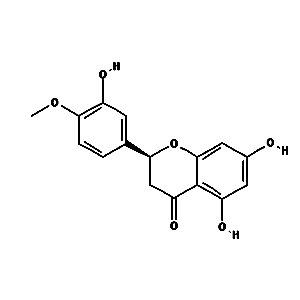 |
| *Glycyrrhiza uralensis* | Isoliquiritigenin | 0.1687 | 0.83 | C_15_H_12_O_4_ | 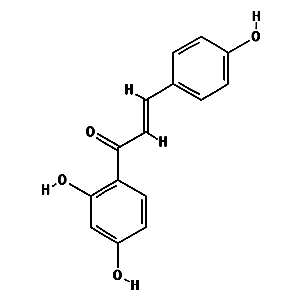 |
| *Angelica sinensis*  *Heracleum hemsleyanum* | Isopimpinellin | 0.03317 | 3.15 | C_13_H_10_O_5_ | 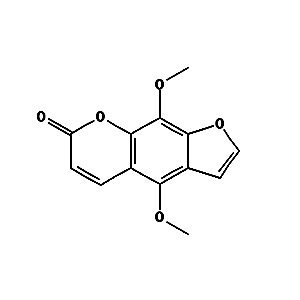 |
| *Heracleum hemsleyanum* | Bergapten | 0.2806 | 0.56 | C_12_H_8_O_4_ | 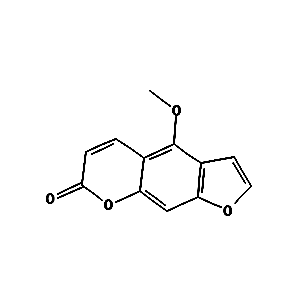 |
| *Citrus reticulata* | sinensetin | 0.3334 | 2.32 | C_20_H_20_O_7_ | 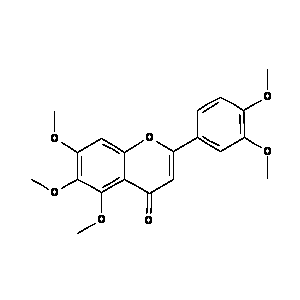 |
| *Citrus reticulata* | Nobiletin | 2.951 | 2.94 | C_21_H_22_0_8_ | 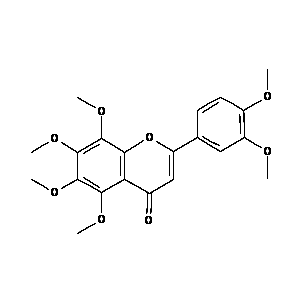 |
| *Citrus reticulata* | Tangeretin | 0.7314 | 3.46 | C_21_H_22_O_8_ | 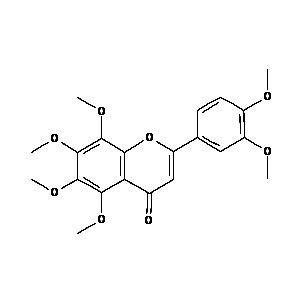 |
| *Citrus reticulata* | 5-Demethylnobiletin | 0.1187 | 1.32 | C_20_H_20_O_8_ | 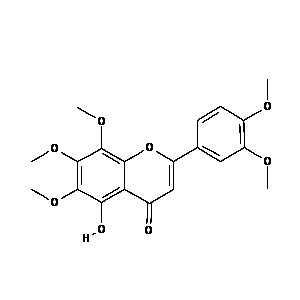 |
| *Heracleum hemsleyanum* | Columbianadin | 1.003 | 0.09 | C_19_H_20_O_5_ | 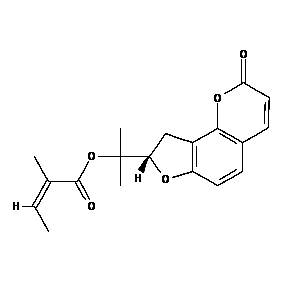 |
